# Archaeometric evidence for the earliest exploitation of lignite from the bronze age Eastern Mediterranean

**DOI:** 10.1038/s41598-021-03544-w

**Published:** 2021-12-17

**Authors:** Stephen Buckley, Robert C. Power, Maria Andreadaki-Vlazaki, Murat Akar, Julia Becher, Matthias Belser, Sara Cafisso, Stefanie Eisenmann, Joann Fletcher, Michael Francken, Birgitta Hallager, Katerina Harvati, Tara Ingman, Efthymia Kataki, Joseph Maran, Mario A. S. Martin, Photini J. P. McGeorge, Ianir Milevski, Alkestis Papadimitriou, Eftychia Protopapadaki, Domingo C. Salazar-García, Tyede Schmidt-Schultz, Verena J. Schuenemann, Rula Shafiq, Ingelise Stuijts, Dmitry Yegorov, K. Aslιhan Yener, Michael Schultz, Cynthianne Spiteri, Philipp W. Stockhammer

**Affiliations:** 1 Institute for Prehistory, Early History and Medieval Archaeology, Eberhard Karls University Tübingen, Burgsteige 11, 72070 Tübingen, Germany; 2 Department of Archaeology, BioArCh, University of York, York, YO10 5DD UK; 3 Institute for Pre- and Protohistoric Archaeology and Archaeology of the Roman Provinces, Ludwig Maximilian University Munich, Schellingstraße 12, 80799 Munich, Germany; 4 Max Planck Institute for Evolutionary Anthropology, 04103 Leipzig, Germany; 5 Excavation House “Sevach”, Kanevaro and Skordilon Str., 73100 Chania, Greece; 6 Archaeology Department, Mustafa Kemal University, Antakya, Hatay, Turkey; 7 Department of Archaeology, University of York, York, YO1 7EP UK; 8 Institute for Archaeological Sciences, Eberhard Karls University Tübingen, Rümelinstrasse 23, 72070 Tübingen, Germany; 9 The Swedish Institute at Athens, Mitseon 9, 117 42 Athens, Greece; 10 Koç University Research Center for Anatolian Civilizations (ANAMED), Istanbul, 34421 Turkey; 11 Ephorate of Antiquities of Chania, Stoa Vardinogianni, 73100 Chania, Greece; 12 Institute for Prehistory, Protohistory and Near Eastern Archaeology, University of Heidelberg, Sandgasse 7, 69117 Heidelberg, Germany; 13 Institute of Archaeology, Tel Aviv University, 69978 Tel Aviv, Israel; 14 Leon Recanati Institute for Maritime Studies, University of Haifa, 3498838 Haifa, Israel; 15 The British School at Athens, Souidias 52, 106 76 Athens, Greece; 16 Israel Antiquities Authority, 91004 Jerusalem, Israel; 17 Ephorate of Antiquities of the Argolid, Syntagma Square, 211 00 Nafplio, Greece; 18 IKERBASQUE-Basque Foundation for Science, Grupo de Investigación en Prehistoria IT-1223-19 (UPV-EHU), 01006 Vitoria-Gasteiz, Spain; 19 Departament de Prehistòria, Arqueologia I Història Antiga, University of València, 46010 València, Spain; 20 Institute of Anatomy and Embryology, University Medical School Göttingen, 37075 Göttingen, Germany; 21 Institute of Evolutionary Medicine, University of Zurich, Winterthurerstrasse 190, 8057 Zurich, Switzerland; 22 Anthropology Department, Yeditepe University, Istanbul, Turkey; 23 The Discovery Programme, 6 Mount Street Lower, Dublin 2, Ireland; 24 Institute for the Study of the Ancient World (ISAW), New York University, New York, USA; 25 Department of Biology, University of Hildesheim, 31141 Hildesheim, Germany

**Keywords:** Mass spectrometry, Biogeochemistry

## Abstract

This paper presents the earliest evidence for the exploitation of lignite (brown coal) in Europe and sheds new light on the use of combustion fuel sources in the 2nd millennium BCE Eastern Mediterranean. We applied Thermal Desorption/Pyrolysis–Gas Chromatography-Mass Spectrometry and Polarizing Microscopy to the dental calculus of 67 individuals and we identified clear evidence for combustion markers embedded within this calculus. In contrast to the scant evidence for combustion markers within the calculus samples from Egypt, all other individuals show the inhalation of smoke from fires burning wood identified as Pinaceae, in addition to hardwood, such as oak and olive, and/or dung. Importantly, individuals from the Palatial Period at the Mycenaean citadel of Tiryns and the Cretan harbour site of Chania also show the inhalation of fire-smoke from lignite, consistent with the chemical signature of sources in the northwestern Peloponnese and Western Crete respectively. This first evidence for lignite exploitation was likely connected to and at the same time enabled Late Bronze Age Aegean metal and pottery production, significantly by both male and female individuals.

## Introduction

**Figure 1:**
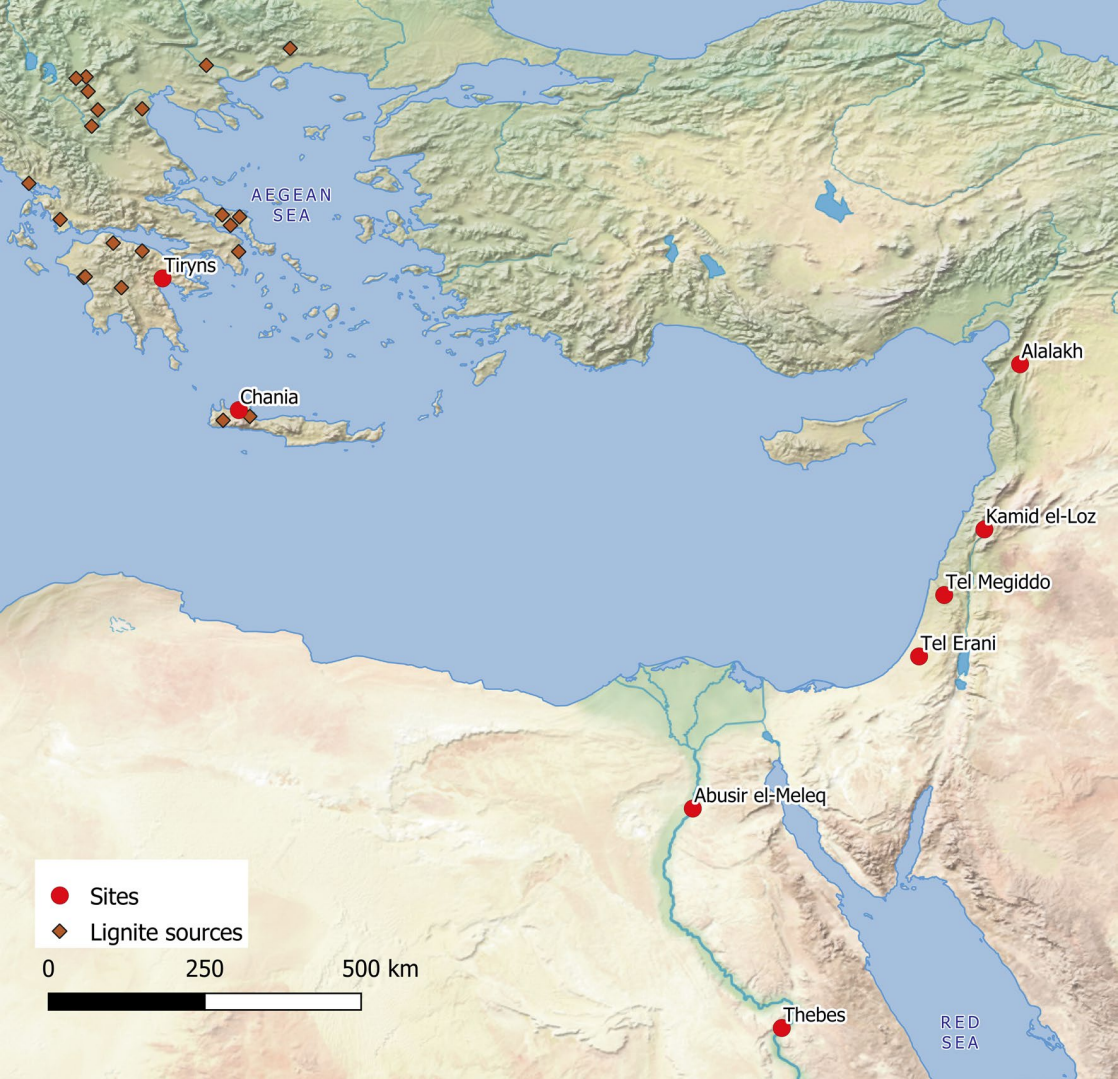
Map of the Eastern Mediterranean featuring the sites included in this study and the currently-known key lignite sources in Greece (created by R.C.P. with QGIS3, using Natural Earth raster data: QGIS.org, 2021. QGIS Geographic Information System. QGIS Association. http://www.qgis.org).

Making fire was a crucial stage in the development of humankind^1,2^ and inhaling its smoke was an inevitable result of this process. The increasing ability to manage fires and their temperatures also allowed for more sophisticated methods of cooking, and enhanced the development of craft technologies such as pottery production and the melting and casting of metals. To date, ancient fire-making has mostly been studied through the residual ash and other remains taken from fireplaces, and from the thermal alteration and soot marks visible on artifacts, in addition to the information provided by experimental archaeology and ethnoarchaeology (e.g.^3–5^). Only recently has it become possible to understand the inhalation of smoke by past individuals by studying its chemical and microscopic traces in human dental calculus^6–9^. Our study of combustion markers in human dental calculus is part of a larger project which aims to understand culinary practices of the 2nd millennium BCE Eastern Mediterranean, i.e. the Middle Bronze Age (ca. 2000–1600 BCE), the Late Bronze Age (ca. 1600–1200/1050 BCE) and the Early Iron Age (after 1200/1050 BCE) (Fig. 1; Table S1; SM Text 2). During this time, the Eastern Mediterranean transformed into an early globalized region, characterized by complex stratified societies employing writing systems and sophisticated craftsmanship as well as large-scale production centres producing goods often aimed at trans-regional exchange. The written and archaeological evidence also reveals a high degree of individual mobility between the Mycenaean Aegean, Hittite Anatolia, Cyprus, the Levant’s trade centres and city kingdoms, Mesopotamia and Middle and New Kingdom Egypt^10–12^. Therefore, we have selected human dental calculus from key sites of the 2nd millennium in the Aegean (Tiryns, Chania), the Levant from the north (Alalakh) via present-day Lebanon (Kamid el-Loz) to the south (Megiddo, Tel Erani) and Egypt (Abusir el-Meleq, Thebes). Tiryns and Chania were major harbour and palatial sites in the Aegean. Tiryns was a focus of large-scale craft production of the Northeastern Peloponnese and closely linked to Mycenae, the nearby major Late Bronze Age political centre in that region of Southern Greece^13^. As part of the increasing control of Crete by the Mainland palaces, Chania became one of the key centres for the Mainland’s exploitation of the island^14^. The Levantine cities (Alalakh, Kamid el-Loz, Megiddo, Tel Erani) were situated in between and usually influenced by or under the control of the great empires of Anatolia, Syro-Mesopotamia and Egypt, the latter represented by samples in our study. The 2nd millennium was also marked by the large-scale production of goods and their subsequent trade, especially pottery and metal objects, throughout the Eastern Mediterranean^10^. Most noteworthy is the mass production of pottery in the Late Bronze Aegean, a large part of which was exclusively produced for export to Cyprus and the Levant. So far, there has been little discussion of the resources that were used to fuel the kilns and ovens for this proto-industrial production in densely settled and probably largely de-forested areas, whereas the fuels used for cooking have been increasingly studied in the Eastern Mediterranean Bronze Age^4,15–17^. Our study of chemical combustion markers aims at a better understanding how this unprecedented level of interconnection transformed both local cooking practices and the procurement of fuel.

## Results

### Chemical analyses

Thermal Desorption-Gas Chromatography-Mass Spectrometry (TD-GC–MS) and Pyrolysis–Gas Chromatography-Mass Spectrometry (Py-GC–MS) of the human dental calculus revealed a significant abundance of combustion markers in 74 of the 77 samples retrieved from the 67 individuals studied (*SM Appendix*, Text 1.2; Tables S1, S2a and S2b).

### Wood and dung combustion

The dominant biomarkers identified were polynuclear aromatic hydrocarbons (PAHs), i.e. organic compounds with two to six membered aromatic rings typical of chars or soot associated with smoke resulting from the long-term and/or repeated exposure to fires in more or less close vicinity. The ratios of these PAHs are typical of combustion, rather than petrogenic sources deriving from modern environmental contamination^18–20^, as would be expected for ancient exposure to smoke/fires. All samples with significant abundances of PAHs also revealed biomarkers indicative of conifer wood combustion. These included the diterpenoid acid esters methyl dehydroabietate and methyl abietate, their free acids being significant components found in Pinaceae wood (which includes genera such as *Pinus*, *Picea*, *Larix*, *Abies* and *Cedrus*) and resin. These thermally-derived methyl esters were accompanied by defunctionalized diterpenoids which included 19-norabieta-4,8,11,13-tetraene, 19-norabieta-4(18),8,11,13-tetraene, 19-norabieta-3,8,11,13-tetraene, tetrahydroretene, dihydroretene, retene and dehydroretene. These methyl esters and defunctionalized diterpenoids reflect the strong heating process which the conifer wood would have undergone during its burning, and its production of the subsequently inhaled smoke. Additional chemical evidence for conifer wood combustion is revealed by the relative abundance of the dimethylphenanthrenes (DMPs). These have been shown to be diagnostic, with 1,7-dimethylphenanthrene, deriving chemically from the diterpenoids characteristic of conifers, dominating the *m/z* 206 profile and producing distinctively high 1,7/1,7 + 2,6-DMP ratios of ~ 0.9^21,22^. The presence of these biomarkers and the characteristic ratios confirms the inhalation of smoke from the burning of conifer (e.g. Pinaceae) woods^23–25^. More generally, the ratios of the PAHs fluoranthene to pyrene have allowed us to further identify and discriminate between dung, wood, and fossil fuel sources, with ratios of ~ 0.8 for dung, ~ 1 for wood and 1.40 for lignite/coal^26,27^. A 1,7/1,7 + 2,6-DMP ratio of ~ 0.5 is typical of animal dung burning, whereas values for oakwood (hardwood) and pinewood (softwood) are ~ 0.7 and ~ 0.9 respectively^18^. The use of dung as fuel was attested in 29 of the individuals studied here, based on the combined 1,7/1,7 + 2,6-DMP ratio consistent with the burning of dung (see SI 1), and a fluoranthene/pyrene ratio of ~ 0.8 indicative of the combustion of dung^26,27^ (Table S1). Hardwoods and softwoods were also used as fuels, the chemical data identifying their use in 58 and 64 individuals respectively (Table S1), and indicating exposure to smoke from multiple fuel sources in the case of the majority of individuals, presumably reflecting local availability of potential fuel resources at these sites.

### Lignite (brown coal) combustion

**Figure 2:**
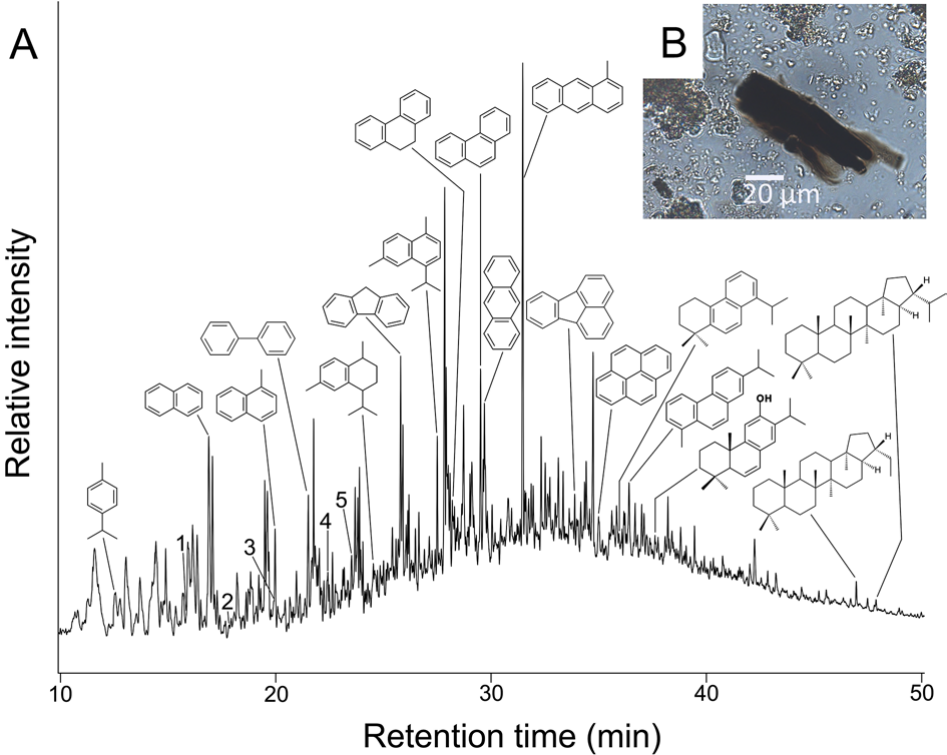
(**A**) Reconstructed total ion chromatogram of the thermal desorption profile (310 °C for 5 min, 610 °C for 10 s (see Materials and Methods)) of human dental calculus from TIR002.B. Peak identities: the lignite/coal markers numbered 1–5 were identified as: 1 = succinimide, 2 = benzoic acid, 3 = benzamide, 4 = phthalic anhydride, 5 = phthalimide. Also shown are the structures of eight polynuclear aromatic hydrocarbons (combustion markers): naphthalene, 1-methylnaphthalene, biphenyl, fluorene, 9,10-dihydrophenanthrene, phenanthrene, anthracene, 1-methyl anthracene, fluoranthene and pyrene. In addition, the structures of the terpenoids characteristic of Cupressaceae deriving from the lignite are shown, i.e. the main monoterpenoid compound identified: p-cymene, the main sesquiterpenoid compounds identified: calamenene and cadalene, and the main diterpenoid compounds identified: diaromatic totarane, retene and 6-dehydroferruginol. The main lignite-derived hopanes, 17α(H),21β(H)-norhopane and 17α(H),21β(H)-hopane are also shown. (**B**) Charcoal particle from TIR002.B (unknown taxon).

An unusual biomarker distribution (Fig. 2) was observed in six Late Bronze Age and two Early Iron Age individuals from Tiryns and three Late Bronze Age individuals from Chania (Table S1). The most diagnostic components included succinimide (m/z 56, 99), benzoic acid (m/z 77, 105, 122), benzamide (m/z 77, 105, 121), phthalic anhydride (m/z 76, 104, 148) and phthalimide (m/z 76, 104, 147) (Fig. 2 and Table S2b). The finding of the succinimide and aromatic biomarkers in samples from Tiryns and Chania and, thus, only in samples from the Aegean, is significant. These specific biomarkers are usually observed together in self-ignited brown and black coal heaps^28,29^ where the potential for self-combustion is high. The absence of these characteristic aromatic biomarkers from the vast majority of the calculus samples, and their co-occurrence specifically in the PAH-rich calculus samples, excludes exogenous, pre- or post-excavation, contamination as a possible source. The presence of these biomarkers is particularly significant since Greece has important lignite deposits, all of which are surface deposits mined as opencast resources^30–34^, which would also have made them visible to ancient populations. The main lignite deposits were formed in intermountain basins such as Ptolemais in Macedonia and Megalopolis in the central Peloponnese, while smaller lignite deposits were created in the western Peloponnese at sites such as Olympia and Pyrgos^30,31^ as well as in Kandanos and Vrysses Apokoronou in western Crete^33,34^. Lignite chemistry reflects the plant input of their formation, which can thereby provide clues to potential sources. The chemical profiles in the calculus samples containing the lignite biomarkers from both Tiryns and Chania do not reveal chemical evidence for sulfur or its derivatives, suggesting the lignite had a low sulfur source. Megalopolis is the closest lignite deposit to Tiryns, although lignite from this site is known to be relatively high in sulfur^31,32^ which is inconsistent with the evidence from Tiryns. Further investigation of the full-scan TD–GC–MS data from the Tiryns calculus samples containing the lignite-related biomarkers also revealed an unresolved complex mixture (UCM), observed as a notable hump in the chromatograms and typical of lignites^35^. Furthermore the biomarkers present are characteristic of higher plants from both gymnosperms and angiosperms consistent with the palaeoecology of southeastern Europe^36^. The gymnosperm-derived diterpenoid compounds dominate the higher plant input, and include not only the fossil biomarkers dehydroabietane, simonellite and 2-methylretene, which can derive from a wide range of conifers, and the more specific 6-dehydroferruginol and diaromatic totarane indicative of a Cupressaceae (e.g. *Taxodium* sp., subfamily Taxodioideae)^37–40^. Importantly, there were no pimarane- or isopimarane-type diterpenoids, nor abietane-type diterpenoid acids detected, which suggests that a significant pine (*Pinus* spp.) input for the lignite can be excluded^37,40,41^. Notably, the diterpenoids identified are also accompanied by a number of monoterpenoids: p-cymene, thujone and carvenone, and the sesquiterpenoids: calamenene, α-calacorene, calamene, cadalene and isocadalene. These terpenoid counterparts to the particular diterpenoids identified have been observed in several Cupressaceae, in particular *Taxodium* spp.^39,42,43^. Lignite from Megalopolis has been noted for its lack of gymnosperms^32^ and an angiosperm predominance^32,36^, which again argues against this as the likely lignite source. The relative abundance of these mono-, sesqui- and diterpenoids is interesting, given the prevalence of conifers growing in a swamp environment, typical of *Taxodium* species and other closely related Cupressaceae (and subfamily Taxodioideae) known to have grown in the western Peloponnese during the late Miocene Epoch^36^. Present-day *Taxodium* and other Taxodioideae are not found in Europe and only in North America. The biomarker evidence for angiosperms includes the presence of 18α(H)-oleanane and it is notable that sample TIR002B shows an *n*-alkane C_max_ at C_29_ for *n*-nonacosane, which is consistent with and indicative of *Quercus* (oak) species, C_29_ known to be the dominant *n*-alkane in *Quercus*^38^. Additionally, the more diagnostic angiosperm biomarkers3,3,7,12a-tetramethyl-1,2,3,4,4a,11,12,12a-octahydrochrysene and 2,2,4ab,9-tetramethyl-1,2,3,4,4a,5,6,14b-octahydropicene^44^, were identified and have been found in angiosperm-containing lignite/coals^45^. Notably, hopanes, most clearly seen in TIR002.B which also has a pronounced UCM indicative of the lignite component, are significant constituents (Fig. 2 and Table S2b); the presence of only trace amounts of hopanes in other lignite-containing Tiryns samples (Table S2b) may reflect the known antibacterial properties of the higher plant terpenoids that dominate these samples^46^. These hopanes are characterized by a base peak at *m/z* 191 and molecular ions (M+.) *m/z* 368, 370, 384, 398, 412, 426, 440, 454, 468 and 482 corresponding to the C_27_, C_28_, C_29_, C_30_, C_31_(S,R), C32(S,R), C_33_(S,R), C_34_(S,R) and C_35_(S,R) 17α(H),21β(H) hopane biomarkers observed (see Table S2a, b for details). The C_29_/C_30_ (norhopane/hopane) ratio of > 1 suggests an anoxic, organic-rich source^47^. Gammarcerane, indicative of a notably saline depositional environment, is absent. The steranes identified in TIR002B (using mass chromatogram *m/z* 217) provide additional detail on the source of the lignite input (see Table S2b). The relative abundances of the ααα-20R C_27_, C_28_ and C_29_ regular steranes was 31%, 38% and 31% respectively. The relative dominance of C_28_ steranes is indicative of a significant algal (e.g. lacustrine) input^48^ and the relative abundance of the regular steranes, combined with the absence of marine-derived C_30_ steranes^47^, suggest a wet environment with terrestrial and planktonic inputs^48^. This is consistent with a paralic-freshwater swamp source for the lignite^48^, as observed for the *Taxodium*-dominated conifer swamps constituting the telmato-deltaic lignite deposits of the western Peloponnese around Pyrgos and Olympia^31,49^. The lignites identified in the three calculus samples from Chania are chemically distinct from the Tiryns lignite source and derive from two different sources (see Table S2b). Chania samples XAN003.B and XAN004.B also revealed biomarkers characteristic of higher plants from both gymnosperms and angiosperms. However, unlike the Tiryns lignite samples these had a far more significant angiosperm input. The common fossil conifer biomarkers dehydroabietane, simonellite and 2-methylretene, and the more specific 6-dehydroferruginol and diaromatic totarane indicative of a Cupressaceae, were not detected in these samples, with retene, dehydroretene and abietic acid (as its thermally-derived methyl ester) being the dominant diterpenoids in these samples and consistent with a Pinaceae input. Although both lignite and wood from extant conifers could be contributing to this, the 1,7/1,7 + 2,6-DMP ratios of 0.5 for both samples would be more consistent with lignite and/or a dung-burning component (13). These were accompanied by the monoterpenoid p-cymene, and the sesquiterpenoids α-cedrene calamenene and α-calacorene, which were also notably different in their relative abundance to those observed in the Tiryns lignite source. Significantly, the samples are dominated by angiosperm biomarkers and are notably high in 18α(H)-oleanane, reflecting this. With an oleanane index (oleanane/hopane) of 1.3 indicative of a very significant angiosperm component in the lignite^50^, an oleanane index above 0.2 also points to a lignite formed in the Tertiary Period^50,51^ and in a marine deltaic environment^51^. Further evidence for a marine input into the lignite, in addition to the gymnosperm and angiosperm terrestrial input, comes from a number of different classes of biomarkers. The pristane/phytane ratio (Pr/Ph) informs on the redox conditions of the depositional environment; low values (< 1) reflect anoxic, deep water and marine inputs^51^, which is consistent with the 0.56 value for these two Chania samples. Hopanes, most clearly seen in XAN004.B which also has a very pronounced UCM indicative of the lignite component, are significant constituents, characterized by a base peak at *m/z* 191 and molecular ions (M+.) *m/z* 368, 370, 398, 412, 426, 440, 454 and 468 respectively, with the C_27_, C_29_, C_30_, C_31_(S,R), C_32_(S,R), C_33_(S,R) and C_34_(S,R) 17α(H),21β(H) hopanes predominating (see Table S2b for details). The C_29_/C_30_ (norhopane/hopane) ratio of > 1 suggests an anoxic, organic-rich source^47^, with the presence of gammarcerane indicative of a saline depositional environment. The steranes identified (using mass chromatogram *m/z* 217) provide additional detail on the source of the lignite input for these two samples (see Table S2b). The short-chain pregnanes, diginane and homodiginane, characterized by a base peak at *m/z* 218 and molecular ions (M+.) *m/z* 288 and 302 respectively, were identified as the major steranes present; these C_21_ and C_22_ biomarkers are notably high in marine, carbonate-rich environments^52,53^ and dominate the C_27_-C_29_ steranes in these samples. Moreover, the relative abundances of the ααα-20R C_27_, C_28_ and C_29_ regular steranes (63%, 22% and 15% respectively) indicate a predominantly marine input, corroborated by the additional presence of the more unusual C_30_ steranes, which are indicative of a marine source^47^. Interestingly, secosteranes, specifically the C_27_, C_28_ and C_29_ 8,14-secosteranes, were identified; these are believed to derive from sponge lipid membranes and their symbiotic bacteria, as previously reported in marine-derived fossil fuels^49^. The decreasing C_31_-C_35_ homohopanes and the diasteranes, being in similar abundance to the regular steranes, also suggests a clastic input for the lignite^54^. In considering the possibility of surface lignite sources local to Chania, Vrysses and Kandanos are in relatively close proximity. Kandanos is an intermontane source and therefore a marine input into the lignite would not be expected. In contrast, Vrysses lignite is within the sedimentary history of the Cretan Neogene as one of frequently changing land-sea distribution^33^. These Neogene sediments have a wide extension in the low coastal plains in the northern part of Chania province. At Vrysses the deposits of Pantanassa Formation show fluvial clastic sediments with paralic and marine sedimentary environments and silty clays, which contributed to the Miocene lignitic beds^33^. There have also been palaeoenvironmental studies of Miocene leaf assemblages from sediments at Vrysses, which have revealed moderate amounts of gymnosperms, including *Pinus* sp., yet angiosperm taxa made up 95% of the total flora^55^. The complex suite of lignitic, marine and terrestrial biomarkers, with *Pinus*-derived diterpenoids and yet a dominance of angiosperm biomarkers, as observed in XAN003.B and XAN004.B, is consistent with the paralic and palaeobotanical context of the lignite source at Vrysses, located only 20 miles away from Chania. In contrast, the lignite source from Chania individual XAN001.B does not contain abietane-type acids, although diterpenoids confirming a gymnosperm conifer input are present. These were accompanied by the monoterpenoid: p-cymene, and the sesquiterpenoids: α-cedrene calamenene, and α-calacorene, although in different relative abundances to XAN003.B and XAN004.B, and included an unidentified methanoazulene, β-cedrene and the tentatively identified sesquiterpene, δ-cadinene, not seen in the samples from these two Chania individuals. Overall, it was again quite different to those terpenoid biomarkers observed in the Tiryns lignite source. There was an absence of both angiosperm and marine biomarkers and was again quite different to those terpenoid biomarkers observed in the Tiryns lignite source. Given the intermontane, inland location, combined with the low sulfur content of the lignite^56^, Kandanos, located only 25 miles from Chania, may have provided this second local lignite source in the case of the XAN001.B individual.

## Microremains

Dental calculus microremains were obtained from 54 individuals, allowing further analysis of combustion-related microremains, including charcoal, non-diagnostic burnt material, unburnt wood remains and melted phytoliths (*SM Appendix*, Text 1.3; Tables S1 & S3; Fig. S2). Although studies of airborne contributions to the composition of dental calculus are still in their infancy and not all populations exposed to smoky environments exhibit combustion products in their calculus^57^, such particles, when present, can reveal local fire use. Variation in abundance and size distribution of these microremains indicates multiple depositional pathways, including food (melted phytoliths, combustion products), airborne transport (combustion products)—mostly within 20 km—and unknown pathways (unburnt wood)^8,58^. For example, while smaller charred particles may reflect inhaled airborne particles transported over long distances, larger combustion particles probably derive from more direct contact with smoky environments, as well as charcoal-covered, charred foods and soil char adhering to food^59^. Charred remains in individuals across samples, together with individuals showing chemical evidence for lignite, tend to contain slightly larger particles (Figs. S2–S7), although given the minimal difference in size it is unclear if this indicates less transport of charred remains or closer contact with fire. Although size-based thresholds for reconstructing transport distances for airborne charred remains in dental calculus have not yet been determined, we note that particles in the 2–10 μm size fraction, PM10, as found at all sites except at Abusir el-Meleq (Table S3), can enter through the throat. nose and subsequently the lungs, and so might be injurious to human health^60^.

### Combustion products

Microparticle analysis revealed black-brown opaque to translucent particles with a size range of 3 to > 100 μm in all samples^61^. Particles smaller than this may be black carbon (amorphous carbon) or black mineral particles and were not quantifiable. Amongst the larger fractions, some were indicative of charcoal, particularly particles ranging from 20 to 60 μm. These can originate from partially combusted wood, straw, leaves, bark and other materials. In most cases, the origin of these combustion particles cannot be identified due to their small size. Identifiable charcoals typically appear as sub-rectangular sheets (Fig. S2), but cannot be attributed to specific taxa when they occur below a size of 100 × 50 μm. The length–width ratios of the particles indicate that they tend to derive from the combustion of timber rather than grass (Table S3). Generally however, the particles present here cannot be identified and for this reason we focus on combustion particles as a whole. These microparticles occur across the samples and they do not appear to be more frequent in certain groups (site, sex etc.) when measured in counts or as per milligram of sample. Neither are they more common in samples that were shown to contain traces of inhalation of lignite smoke. They occur in a highly variable pattern, from several particles to hundreds in each sample. They are rare in several samples, including ABU003, XAN008 and MGD008.

### Lignite combustion

Lignite reference samples from Ptolemais (Greece) and Staniantsi (Bulgaria) were analyzed to identify morphology diagnostic of lignite. Lignite particles were found to be opaque and exhibited little plant cell morphology. Microscopic analysis showed that although lignite morphology largely overlaps with charcoal, it is distinctively uniformly fractured. However, the identification of lignite fragments under the polarizing microscope requires large particle sizes for identification, which are usually not embedded in dental calculus. Possible fragments in the calculus are too small to distinguish confidently.

## Discussion and conclusion

The chemical data obtained in this study show the first and earliest evidence obtained to date for the exploitation and use of lignite in Europe and the Mediterranean during the 2nd millennium BCE. The GC–MS and microscopy have also further demonstrated the diversity of fuels being used at this time in the Eastern Mediterranean, and we were able to link the usage of fuel with particular individuals via their inhalation of combustion markers.

With regard to the selection of fuel for fire-making, it seems evident that burning conifer (Pinaceae and Cupressaceae spp.) wood, or charcoal made from this wood, played a crucial role in fire management. The distribution maps of wood species in the area suggest that *Pinus nigra*, *Pinus pinea* and *Abies cephalonica* found widely across the Eastern Mediterranean would have been the most likely conifer sources^62,63^. Moreover, our data shows that hardwood like oak and probably olive was also used, albeit on a seemingly slightly less frequent basis. The virtual absence of wood combustion markers in samples from Egypt is likely due to the fact that the individuals studied were not exposed to open fires for long periods of time, perhaps due to a consistently warmer climate. Although recent research has pointed to the importance of dung for fire-making in the prehistoric past^64–67^, its use has rarely been considered for the 2nd millennium BCE Eastern Mediterranean, but our results suggests it seems to have been an important additional fuel in almost all contexts. This evidence points to an opportunistic use of locally available wood and dung for daily fire making in the context of cooking, heating, torches, incense burners etc. The occurrence of wildfires might also have had an impact, albeit on a much smaller level compared with the regular and daily exposure to domestic fires. An integrative analysis of contextual, archaeobotanical, phytolith and anthracological studies of ovens in pyrotechnological workshops at Tiryns has indicated the relevance of *Pinus nigra*, *Abies* sp. and *Quercus* sp. for fire-making, including *Olea europaea*, consistent with the data presented here and indicating the use of softwoods and hardwoods as fuel sources. Inhalation of combustion particles from the burning of *Olea europaea* could not be differentiated beyond the burning of hardwoods.

Most notably and unexpected is the evidence for the combustion of lignite. So far, lignite was thought to have been used for the first time in Classical Greece, as discussed by Theophrastus (374/369–288/285 BCE) when describing different kinds of coals with regard to fire and temperature management (Theophrastus, On Stones, 16; Translation:^68^). He also mentions the possibility that lignite could have been collected from the surface of the land close to Olympia (i.e. in the western Peloponnese; ca. 150 km overland distance from Tiryns), and that lignite was particularly useful for metallurgy. Clearly this literary source demonstrates ample experience with lignite as a resource for fire management by the fourth century BCE, but it had not been previously possible to date the beginning of its exploitation or confirm its technological use scientifically. Although recently published evidence suggests lignite was being used in China by ca. 1600 BCE^69^, it seems that its exploitation in Greece had started in the thirteenth century BCE at the very latest, given that the Tiryns individuals with chemical evidence for lignite in their dental calculus lived and worked during the decades between the mid-thirteenth century and the early twelfth century BCE, i.e. the heyday of the Mycenaean palatial workshops at Tiryns and its immediate aftermath. The individuals from Chania are slightly earlier and belong to the fourteenth-thirteenth centuries BCE, when Chania was under Mycenaean control after Crete had been conquered by forces from the Greek mainland during the fifteenth century BCE and Crete was subsequently increasingly exploited^70,71^.

There is no clear link between the inhalation of lignite particles and sex, and both sexes seem to have been involved in lignite pyrotechnologies. All individuals showing lignite particles from the Lower Citadel of Tiryns were buried with no additional goods, which has been interpreted as an indication of their lower status, whereas their placement under or between the high-status architecture in this area contradicts this interpretation^72^. All three individuals with chemical evidence for lignite inhalation from Chania and two from Tiryns (TIR002, TIR006) also show anthropological evidence for hard physical labor, with a pronounced physical development of the muscles of the right arm. One female individual from Chania (XAN001) even shows evidence of degenerative/rheumatic changes, whereas the other two individuals with lignite inhalation probably died too young to display these changes^73^. Although it is challenging to link this anthropological evidence to specific kinds of labor, these individuals might nevertheless be interpreted as male and female craftspeople of probably different social statuses, who at least in part, conducted heavy work close to the kilns and ovens that produced pottery, metal and/or vitreous materials.

Tiryns, Chania and their surroundings were important centres for the large-scale production of pottery during the Late Bronze Age. The landscape of the Argolid around Tiryns was known for its large-scale pottery production. This is evident from specialized potters’ centres like Berbati^74^, the pottery kiln from the early twelfth century BCE found on the Lower Citadel of Tiryns in the immediate vicinity of the slightly older burials we studied^75,76^, and the tens of thousands of vessels which seem to have been produced every year to meet the demands of the palaces and beyond, given that they were exported all over the Mediterranean^77,78^. Moreover, there is evidence for metal working in Tiryns in the thirteenth and twelfth centuries BCE^79,80^ as well as for glass working^81^. For Chania, there is ample evidence for the large-scale production of fine ware pottery and large transport vessels in the region^82^. The limitation of locally available wood resources must have posed significant challenges, since it is highly likely that densely settled areas like the Late Bronze Age Argolid or the bay of Chania were largely deforested at that time. Such heat-intensive craft production must have raised the need to acquire sufficient additional fuel, with lignite being particularly useful due to the higher energy density and easier temperature control^83^. Moreover, at Chania, it seems natural for lignite to have been sourced from deposits in close proximity, at both Vrysses and Kandanos, but in the case of Tiryns, it took a particular effort to transport lignite from deposits at Olympia over ca. 150 km by land, and even further by sea. This underlines the scale and capability of Mycenaean palatial administration which is well known from textual sources of that time^84,85^. It is also interesting to note the lack of evidence for the exploitation of the sulfur-rich lignite deposits around Megalopolis in the central Peloponnese (ca. 80 km overland distance from Tiryns), despite their greater distance from Tiryns. This may point to knowledge of the adverse effects of sulfur in metallurgy. The systematic exploitation of lignite, possibly to supply the high fuel demands of the workshops, would have contributed to the establishment of permanent, large-scale specialized craft production at palaces, which were usually associated with workshops^86,87^, or as specialized craftspeople villages like the aforementioned village of Berbati, irrespective and detached from local wood resources. The possibility of establishing permanent fuel-intensive manufacturing places with the help of coal would later become most prominent and evident with modern industrialization^88^.

Future research will reveal if similar specialized pyrotechnologies can also be traced at other sites, and what proportion of the overall populations at both Tiryns and Chania were exposed to the inhalation of smoke from lignite fires, a fuel type that would later become both central and integral to the modern industrial revolution some 3000 years later.

## Methods

### Archaeological background

Intensive archaeological fieldwork in the Eastern Mediterranean has brought to light a rich corpus of human skeletal evidence from the 2nd millennium BCE (Fig. 1; *SI Appendix*, Text 2; Table S1). All individuals selected by us for analysis were excavated and documented in their archaeological context, most of them found in different kinds of intramural burials. All burials were studied anthropologically, palaeopathologically using medical techniques in the case of Tiryns, and archaeologically; dating is based on radiocarbon dates and/or associated grave goods.

### Chemical analyses

Eighty samples of dental calculus taken from 67 human individuals from eight sites across the Eastern Mediterranean were analyzed by Thermal Desorption-Gas Chromatography-Mass Spectrometry (TD–GC–MS) and Pyrolysis–Gas Chromatography-Mass Spectrometry (Py–GC–MS). Two sediment samples from one of the sites (Alalakh: ALA119.C), were additionally analyzed. This technique facilitates the identification of both free/unbound and bound/polymeric organic components. It also involves minimal sample manipulation which reduces the problems of contamination and sample loss, and requires small sample sizes^89,90^, which is often an important consideration with archaeological material. All 77 calculus samples produced moderate to significant amounts of free organic material. They also revealed similarly moderate to significant amounts of a bound/polymeric organic constituent.

Polycyclic aromatic hydrocarbons (PAHs) were of key interest in this study, hence the need to enhance their detection as part of the methodology applied. Characterization of PAHs provides some concerns in terms of the instrumental parameters used during analysis, namely the need to release ancient PAHs from the matrix they are adsorbed onto without creating them as artefacts of thermal cracking^91,92^. In this case, the impact of the substrate is minimal, given that the calcium in the hydroxyapatite matrix of the dental calculus does not promote the formation of PAHs^93^ Furthermore, to produce PAHs from thermal cracking a minimum temperature of c. 700 °C is required^93,94^, which is significantly higher than the temperatures reached here, preserving the signatures introduced in antiquity. The instrument parameters used for this study were selected on the basis of previous work carried out on optimizing PAH detection and analyses^92,93,95,96^.

TD/Py-GC–MS was performed on a Gerstel TDU Cryogenically-controlled Pyrolysis unit interfaced (320 °C), to an Agilent Technologies 7890B GC split/splitless injector (280 °C) linked to an Agilent Technologies 5977A MSD (electron voltage 70 eV, filament current 220 μA, source temperature 230 °C, quadrupole temperature 150 °C, multiplier voltage 2200 V, interface temperature 300 °C). The acquisition was controlled by a MassHunter ChemStation computer, in full scan mode (10-550amu). Approximately 2–5 mg of calculus sample was weighed into a quartz tube with glass wool end plugs. The tube was placed into a pyroprobe platinum heating coil via a Gerstel Multi-Purpose-Sampler and Gerstel Cold-Injection-System 4. The sample was thermally desorbed at 310 °C for 5 min in open split mode at 60 mL/min. After 4.5 min, the sample was pyrolyzed at 610 °C for 10 s, to provide sufficient energy for higher molecular weight PAHs to become volatilized and detected, but for a short enough time to prevent complete pyrolysis or the formation of PAHs in situ from macromolecular material^93,94^. At the same time the GC temperature program and data acquisition commenced. Separation was performed on a fused silica capillary column (30 m × 0.25 mm i.d) coated with 0.25 μm 5% phenyl methyl silicone (DB-5). Initially the GC was held at 45 °C for 5 min and then temperature programmed from 45 °C-320°C at 6 °C/min and held at final temperature for 15 min, total 65.83 min, with helium as the carrier gas (constant flow 1 mL/min, initial pressure of 7.3614 psi, split at 60 mL/min). The run was repeated with the same sample being pyrolysed at 610 °C for 20 s. Peaks were identified on the basis of both their mass spectra (NIST Mass Spectral Database and additional data referenced below), and relative retention times (relative retention indices (RRIs)).

The findings of the chemical analysis are shown in Tables S1, S2a and S2b.

### Analysis of microremains (*SI Appendix*, Text 1.2)

Extraction used an EDTA-based approach as it poses fewer vapor risks compared with HCI methods^97^. We added ~ 1 ml of 0.5 M EDTA to decalcify weighed dental calculus chunks in 1.5 ml eppendorf tubes under a Bio Air Aura Mini laminar flow in the Department of Primatology at the Max Planck Institute for Evolutionary Anthropology, Leipzig. Samples were left in EDTA until decalcification was complete, which varied from a few hours to a few days. The samples were then centrifuged at 2000 × g for 10 min (Roth Mini-centrifuge), and the EDTA was removed from the samples by pipetting the supernatant. This process was repeated three times. During the final wash, 25% glycerine was added. In some cases, decalcification was already complete from prior protein extraction. In these cases, 100 μl of 25% glycerine solution was directly but slowly added to the tubes to avoid spillage due to foaming of residual sodium dodecyl sulfate from protein extraction. When samples were ready, 20 µl of each sample were mounted on glass slides with 18 × 18–22 × 22 mm coverslips. Mounting was done in the laminar flow and examined under brightfield and cross-polarized light on a Zeiss Axioscope microscope at 400 × magnification (Num. Aperture = 0.95). Samples were analyzed by examining the whole slide and any encountered microremains were photographed, described, and documented using the International Code for Starch Nomenclature (ICSN, 2011) and International code of Phytolith Nomenclature^98,99^. For combustion products, length and width measurements were taken to further identify the source and this was done using micrographs (Measurement data presented in Table S1 and S3).

## Supplementary Information

Supplementary Information 1.

Supplementary Information 2.

Supplementary Information 3.

Supplementary Information 4.

Supplementary Information 5.
