# Supplementary material for: Archaeometric evidence for the earliest exploitation of lignite from the bronze age Eastern Mediterranean
Source: Sci Rep. 2021 Dec 17;11:24185. doi: 10.1038/s41598-021-03544-w (PMC8683508; doi:10.1038/s41598-021-03544-w)
Supplement: Supplementary file 1 — Supplementary Information 1. [file 41598_2021_3544_MOESM1_ESM.pdf]

Supplementary Information Appendix

Archaeometric Evidence for the Earliest Exploitation of Lignite from the Bronze Age Eastern Mediterranean

Authors: Stephen Buckley[1,2]*, Robert C. Power[3,4], Maria Andreadaki-Vlazaki[5], Murat Akar[6], Julia Becher[1], Matthias Belser[1], Sara Cafisso[1], Stefanie Eisenmann[7], Joann Fletcher[8], Michael Francken[9], Birgitta Hallager[10], Katerina Harvati[9], Tara Ingman[11], Efthymia Kataki[12], Joseph Maran[13], Mario A. S. Martin[14,15], Photini J.P. McGeorge[16], Ianir Milevski[17], Alkestis Papadimitriou[18], Eftychia Protopapadaki[12], Domingo C. Salazar-García[19,20], Tyede Schmidt-Schultz[21], Verena J. Schuenemann[9,22], Rula Shafiq[23], Ingelise Stuijts[24], Dmitry Yegorov[17], K. Aslıhan Yener[25], Michael Schultz[21,26], Cynthianne Spiteri[1]*, Philipp W. Stockhammer[3,7]*

Affiliations:

[1] Institute for Prehistory, Early History and Medieval Archaeology, Eberhard Karls University Tübingen, 72070 Tübingen, Germany.

[2] BioArCh, Department of Archaeology, University of York, YO10 5DD, United Kingdom.

[3] Institute for Pre- and Protohistoric Archaeology and Archaeology of the Roman Provinces, Ludwig Maximilian University Munich, 80799 Munich, Germany.

[4] Max Planck Institute for Evolutionary Anthropology, 04103 Leipzig, Germany.

[5] Excavation House "Sevach", Kanevaro and Skordilon Str., 73100 Chania, Greece.

[6] Mustafa Kemal University, Archaeology Department, Antakya, Hatay, Turkey.

[7] Max Planck Institute for Evolutionary Anthropology, 04103 Leipzig, Germany.

[8] Department of Archaeology, University of York, York, YO1 7EP, United Kingdom.

[9] Archaeological Sciences, Eberhard Karls University Tübingen, Rümelinstrasse 23, 72070 Tübingen, Germany.

[10] The Swedish Institute at Athens, Mitseon 9, Athens, 117 42, Greece.

[11] Koç University Research Center for Anatolian Civilizations (ANAMED), Istanbul, 34421, Turkey.

[12] Ephorate of Antiquities of Chania, Stoa Vardinogianni, Chania 73100, Greece.

[13] Institute for Prehistory, Protohistory and Near Eastern Archaeology, University of Heidelberg, Sandgasse 7, 69117, Heidelberg, Germany.

[14] Institute of Archaeology, Tel Aviv University, Tel Aviv 69978, Israel.

[15] Leon Recanati Institute for Maritime Studies, University of Haifa, Haifa 3498838, Israel.

[16] The British School at Athens, Souidias 52, Athens 106 76, Greece.

[17] Israel Antiquities Authority, Jerusalem 91004, Israel.

[18] Ephorate of Antiquities of the Argolid, Syntagma Square, Nafplio 211 00, Greece.

[19] IKERBASQUE-Basque Foundation for Science, Grupo de Investigación en Prehistoria IT-1223-19 (UPV-EHU), 01006 Vitoria-Gasteiz, Spain.

[20] Departament de Prehistòria, Arqueologia i Història Antiga, University of València, 46010 València, Spain.

[21] Institute of Anatomy and Embryology, University Medical School Göttingen, 37075 Göttingen, Germany.

[22] Institute of Evolutionary Medicine, University of Zurich, Winterthurerstrasse 190, 8057 Zurich, Switzerland.

[23] Anthropology Department, Yeditepe University, Istanbul, Turkey.

[24] The Discovery Programme, 6 Mount Street Lower, Dublin 2, Ireland.

[25] New York University, Institute for the Study of the Ancient World (ISAW), New York, USA.

[26] Department of Biology, University of Hildesheim, 31141 Hildesheim, Germany.

Correspondence to: stephen.buckley@uni-tuebingen.de; cynthianne.spiteri@uni-tuebingen.de; philipp.stockhammer@lmu.de

This PDF file includes:

Supplementary Text……………………………………………………………4
Supplementary Figures S1 to S9…………………………………………………19

Other Supplementary Materials for this manuscript include the following:

SI Table S1: Information on the archaeological context and the scientific results obtained for individual samples

SI Table S2a: Chemical information on the presence/absence of combustion-related organic compounds/biomarkers in all individual samples in this study.

SI Table S2b: Chemical information on the quantitative data for combustion-related organic compounds/biomarkers of those samples containing evidence for lignite (brown coal).

SI Table S3: Dimensions of all combustion product particles for individual samples

1. Materials and Methods

1. Analytical Protocol

The data presented here is part of the larger ERC Starting Grant project "FoodTransforms" that applies a multi-method approach to the study of human dental calculus in order to trace past individual culinary practices. Given the small quantities and the heterogeneous nature of dental calculus, an analytical pipeline was established to maximize data collection from the various scientific methodologies used (aDNA, palaeoproteomics, lipid biomarker analysis, and plant microremains). Two main criteria were established based on i) the size and amount of the dental calculus present in each individual, and ii) the quality of the data obtained from the various methodologies. In this respect, it was not always possible to apply both chemical and plant microremains analyses to each individual. Chemical analysis was prioritized when the amount of dental calculus from the individuals was low. Pilot studies also showed the possibility of analyzing plant microremains after protein extractions [1]. This was carried out where possible to maximize data collection.

1.2 Chemical Analysis

Seventy-seven samples of dental calculus taken from 67 individuals from eight sites across the eastern Mediterranean were analyzed by Thermal Desorption-Gas Chromatography-Mass Spectrometry (TD-GC-MS) and Pyrolysis-Gas Chromatography-Mass Spectrometry (Py-GC-MS). This technique facilitates the identification of both free/unbound and bound/polymeric organic components. It also involves minimal sample manipulation which reduces the problems of contamination and sample loss and requires small sample sizes [2,3], which is often an important consideration with archaeological material. All 77 calculus samples produced moderate to significant amounts of free organic material. They also revealed similarly moderate to significant amounts of a bound/polymeric organic constituent.

The methodology and instrumental conditions are detailed in the main text. The findings of the chemical analysis are shown in Tables S1, S2a and S2b.

In order to understand possible contamination issues, we analyzed two control samples from Alalakh, i.e. sediment excavated close to the mandible from which the calculus was analyzed (see Tab S1). These control samples (sediments) were dominated by a narrow range of *n*-alkanes (C19 to C30, maximizing at C21), with the polynuclear aromatic hydrocarbons (PAHs) indicative of combustion (presumed to be from ancient wildfires) present only as very minor constituents. In contrast, the calculus was dominated by mid-chain alkenes (C13 and C14), indoles and moderate amounts of *n*-alkanes (C22 to C33, maximizing at C23), with a wider range of PAHs and the conifer biomarker retene in significant abundance. Notably, the key combustion ratios (see Table S1) of the control (sediment) samples were very different to the combustion ratios of the calculus, reflecting their very different origins and indicating that the integrity of the calculus and the ancient biomolecules contained within the inorganic matrix has been maintained, as observed in previous studies [4].

1.3 Microremains

Introduction to non-dietary particles in dental calculus. Although primarily comprising calcium phosphate and biofilm molecules, research has shown that dental calculus is a rich source of dietary and environmental debris that are present in the oral cavity [5–9]. Recent studies in archaeological science have predominantly focused on dental calculus as a record of

food remains. However, it is also known to contain non-dietary particles related to life history such as char, pollen and diatoms. Some of the earliest dental calculus studies have already drawn attention to their significance [10]. Char and charcoal are commonly studied microremains used in archaeology and paleoecology for understanding the use of fire. Although typically studied in sediments, it has also been recently identified as a component of dental calculus microremain assemblages [4,11,12]. Yet the processes that allow non-dietary particles to become trapped in dental calculus are still not sufficiently understood [7,13], including the mechanisms leading to combustion particulates becoming embedded in this substrate, which has the potential to provide a wealth of information on human-fire interactions.

Analysis of Microremains. Microremains were extracted from dental calculus samples from 55 of the individuals. Extraction used an EDTA-based decalcification approach as it poses fewer vapor risks to human health compared with HCl methods [14]. Around 1 ml of 0.5 M ready dissolved EDTA was added to decalcify previously weighed raw dental calculus chunks in 1.5 ml Eppendorf tubes. This was carried out under a Bio Air Aura Mini laminar flow cabinet in the Department of Primatology at the Max Planck Institute for Evolutionary Anthropology, Leipzig. Samples were left in EDTA until decalcification was complete, which varied from a few hours to a few days. A record was kept of the time it took each sample to decalcify. The samples were then centrifuged at 2000 x g for 10 minutes (Roth Mini-centrifuge), and the EDTA was removed by pipetting the supernatant. This process was repeated three times. During the final wash, 25% glycerine was added. In some cases, decalcification was already complete after a separate and prior protein extraction. The protein extraction protocol applied has been reported to not damage microremains, which were recovered as pellets [15] (also see below). In these cases, 100 µl of 25% glycerine solution was directly but slowly added to the tubes to avoid spillage due to the foaming of residual sodium dodecyl sulfate from the protein extraction. When possible, sediment found close to the cranial part of the skeleton was analyzed to cross-check their microremain content with those found in the dental calculus samples. This cross-checking was, however, limited by the availability of relevant sediment (n=2), as this was usually not kept during fieldwork which in most cases was carried out years before the planning of this project. Sediment controls received identical EDTA treatment.

Microremains extraction from chunks of dental calculus vs. pellets from a prior protein extraction. Combustion particles were extracted from raw dental calculus chunk samples and from residual pellet samples processed via proteomic extraction. Care was taken to test that the different methodologies did not affect the number and size of the combustion particles extracted. To assess if the two different extraction methods (raw chunks and residual pellets) yielded different results, we compared the total number of combustion particles in raw samples and residual pellet samples from protein extractions and the length and width ratios of the combustion particles (Fig. S1). These comparisons show that these treatments yield similar numbers of particles and that dual protein-microremain extraction has little impact, consistent with our expectations.

Reference particles and archaeological particle identifications. On rare occasions, phytoliths exhibited a dark carbon coating (as opposed to carbon inclusions observed in the core of grass short-cells), and these were identified as burnt phytoliths. These phytoliths were discolored but

not very dark, and were sometimes misshapen or had a bubbled surface. These were interpreted as phytoliths partially melted during exposure to ancient combustion [16].

Particles that were observed to be black-brown and opaque-translucent with sharp geometric edges (Fig. S2a–c) were deemed 'probable combustion products' [16–19]. Often, these particles are more prominent than phytoliths due to their dark brown to black opaque color that contrasts with the transparent colorless nature of phytoliths and pale brown clay aggregations. Combustion products include charcoal produced from burnt wood, bark, shell and grasses but it also includes amorphous black carbon. Some particles exhibited remnant wood morphology ie. vascular systems that indicated that they were charcoal, but in most cases we did not advance origin above the category of combustion particle (Fig. S2a–c). Remnant wood morphology is used by anthracologists to identify the tree species of the resulting charcoal. However, this could not be achieved within this study due to the small particle size [20]. The prevalence of these combustion particles is highly variable in terms of absolute numbers per sample and per mg of sample. Overall, these combustion particles do not occur more frequently in the different regions of the study, but high concentrations are observed in a number of the Megiddo and Tel Erani samples (>150 per a sample). Combustion particles were infrequent in all Chania samples analyzed for microremains. Although they were sometimes highly prevalent at Megiddo, this trend is driven by a small number of samples that had a high initial mass prior to decalcification (e.g., MGD02; Data presented in Table S1). Other samples had very low numbers of combustion particles (<15 per a sample) and some had no examples at all.

Length and width ratios were calculated to explore their origin (Table S3), i.e. to understand if these particles tend to originate from grass (> 1:0.5) or wood (1:< 0.5) sources [21]. Grass charcoal is often environmental in origin and is less likely to represent human burning activities.

Reference lignite samples from Greece and Bulgaria were similarly analyzed. Results showed that the lignite particles have very low length and width ratios, and were also larger in size, more opaque and more angular than charcoal, and had distinct fracturing (Fig. S2d). These diagnostic criteria, however, apply to large pieces of reference lignite particles, typically 100 microns or more in diameter. Large combustion products are rare in dental calculus, and features such as the fracturing pattern cannot be securely assessed for the typically small particles present here. For this reason, a confident identification of lignite could not be achieved via the microscopy in this study. We tried a metric-based identification (described below), but at this stage, we could not define diagnostic criteria for lignite that are widely applicable.

Metric-based identifications. To assess the possibility of finding distinct patterns in the larger particles that might indicate the presence of lignite, we compared the abundance of combustion products, and the mean length and width of the particles across the samples. The results were combined when raw dental calculus chunks and residual pellets from previous protein extractions were available from the same sample. In cases when multiple dental calculus samples were analyzed per individual, the results were grouped as a single entity. We used this approach to test associations of chemical evidence for lignite with sex, site, and for male and female individuals in Tiryns in particular, who showed significant chemical evidence for lignite (Fig. S3–6). Our results showed that particle size was not sufficiently distinct to securely differentiate lignite from other combustion particles.

We compared size distribution to assess if there are differences between samples with chemical evidence for lignite, and samples lacking this signal (Fig. S3). No difference between

the groups was identified, implying that lignite could not be distinguished from the particles identified as combustion particles.

Possible sex-related differences were analyzed by comparing the combustion product dimensions in males with females. This was compiled as a whisker plot that divided the data into quartiles with an exclusive median for the length and width (Fig. S4). We divided individuals into male and female individuals. This comparison excluded individuals with unclear biological sex determination. No distinguishing patterns were identified.

The length and width of all combustion particles from each site investigated were plotted in a separate whisker plot (Fig. S5). This was carried out to test for peculiar patterns that might indicate the presence of lignite. Tiryns presented some of the largest combustion particles, which would have been significant given the pronounced chemical evidence for lignite identified here, but overall no distinguishing features in the particles identified as combustion particles could be made. Thus, combustion particle size at Tiryns does not show a distinct pattern, and variation in the graph is driven by sample size. Combustion particle size from male and female individuals at Tiryns were plotted in a whisker plot (Fig. S6) to attempt to obtain a better perspective on the size of the combustion particles, although we found no differences between the sexes. For the Tiryns samples, we explored if the number of combustion particle fragments show a distinct pattern in particular individuals that could indicate lignite use, and if some of the detected combustion particles are in fact lignite particles. However, once again no meaningful correlation was observed.

Sediment controls. Comparing the number of combustion particles in dental calculus (n=18) versus sediment samples (n=2) from Alalakh revealed that these two materials had very different assemblages. The sediment was a richer source of combustion particles (Fig. S7), which were larger (mean: 27.5 microns in length, 12.9 microns in width) than in the calculus (mean: 18 microns in length, 11.4 microns in width). This suggests that the charcoal in calculus samples could have a different origin rather than be due to post mortem contamination; however, it could also result from selection biases that favor the inclusion of smaller particles in the dental calculus, although it is not clear if this can actually occur post mortem. Unfortunately, no sediment samples were kept during fieldwork related to the other samples analyzed in this study.

Identification of other particles. The analyses also explored other particles relevant to the study of combustion and fuel (Fig. S2). Transparent sheets of tissue that might be identified as unsilicified plant tissue, including wood tissue, were documented. Remnant wood structure such as vascular morphology, e.g. bordered pitting of tracheids, was used to characterize wood taxonomy into the lowest possible taxonomic units, including one conifer fragment and one cf. *Alnus* fragment, both showing no evidence for burning (Fig. S2e–f) [17,22].

Summary

Combustion products and charcoal can occur in many size ranges. In this study, only small particles were found in the dental calculus samples tested. We found larger examples in the sediment samples which might indicate different sources. While combustion particles in the sediment probably represent local conflations, possibly *in situ*, particles in dental calculus are consistent with windblown charcoal, probably over long distances, and local charcoal sources such as the inhalation of smoke from nearby fires, e.g. related to heating or cooking practices or through transference in food and water. Size distribution of the particles across the samples is

largely unremarkable. In samples with larger numbers of particles (Megiddo and Tiryns), greater variation was identified. In addition to combustion particles, occasional unburnt wood is present.

Exposure of the individuals to combustion particles is also evident from a small number of burnt phytoliths, possibly indicating higher temperatures (see Table S1) [16]. However, phytoliths that showed melting during combustion at high temperatures were a rare component of the assemblages compared with combustion particles.

2. Archaeological Contexts

Intensive archaeological fieldwork in the Eastern Mediterranean has brought to light a rich corpus of human skeletal evidence from the 2nd millennium BCE (Fig. 1; SI Appendix, Text 2; Table 1). All individuals selected by us for analysis were excavated and documented in their archaeological context, most of them found in different kinds of intramural burials. All burials were studied anthropologically, paleopathologically using medical techniques in the case of Tiryns, and archaeologically; dating is based on radiocarbon dates and/or associated grave goods.

2.1 Tiryns, Greece (sample names "TIR")

The site of Tiryns, with its Citadel and Lower Town, was one of the major palatial centers of the Mycenaean period in Greece between ca. 1400 and 1200 BCE, and the most important harbour of the Peloponnesian Argolid region [23]. Compared to other sites, Tiryns recovered much more rapidly from the setback caused by the destruction of the palace around 1200 BCE, becoming one of the foremost centers of the ensuing post-Palatial Period within the Aegean (ca. 1200–1050 BCE) [24,25].

Excavations in the Lower Citadel and Lower Town of Tiryns, directed by Nikolaos Verdelis (1965), Ulf Jantzen (1968–1974), Klaus Kilian (1976–1983), Joseph Maran (2001–2003) and Joseph Maran and Alkestis Papadimitriou (2013–2018), have uncovered burials dating to the time span between the Mycenaean and Byzantine periods. The ongoing anthropological-paleopathological study of the human skeletal remains from these burials by Michael Schultz and Tyede H. Schmidt-Schultz began in 2011. It aims to determine the sex, age, body height, type of constitution and handedness, in addition to finding evidence of living conditions, diseases and, where possible, the cause of death, in order to demographically characterize this segment of the Tiryns' population [26]. The skeletal individuals identified were differentiated by the anthropologists using a numbering system consisting of the name of the excavator and a consecutive number starting with "1" for each excavator (cf. "Kilian 35"). Hitherto, at least 150 skeletal individuals dating to the Palatial or post-Palatial Period were identified among those uncovered in excavations since the 1960s.

The skeletal individuals from Tiryns included in this project come from excavations in the Lower Citadel and date to the Mycenaean Palatial and Post-Palatial period (TIR001–006, TIR010, TIR014–018). With the exception of the sample TIR003 that comes from a burial furnished with several ceramic vessels [27], all other Mycenaean samples derive from burials without grave goods. Such single burials and groups of burials *intra muros* without grave goods mostly dating to the late Palatial Period (ca. 1250–1200 BCE) and the early post-Palatial Period (ca. 1200–1150 BCE) [23] have been uncovered since the 1960s, in different parts of the Lower Citadel. Despite the lack of grave goods, the burials do not have an irregular or haphazard

appearance, since the deceased were carefully deposited on their backs in an extended position or lying on their side in a crouched (ie. 'foetal') position in shallow pits, in open areas or within the ruins of houses.

The burials without grave goods in the Tirynthian Lower Citadel markedly deviate from the Palatial and post-Palatial Period burial habit of interring the dead with grave goods in chamber tombs dug into the slopes of hills in the surroundings of a settlement [28]. Kilian [29] interpreted such burials as those of members of low social status groups within the palatial society to whom the right to be buried with grave goods in such chamber tombs had been denied. However, the practice of depositing the dead without grave goods continued after the destruction of the palace, when the social conditions must have been considerably different to those from the Palatial Period. In the early 12th century BCE it seems that the entire northernmost part of the Lower Citadel was temporarily transformed into a burial ground for such burials [30]. Therefore we may be dealing with funerary traditions that were not only practiced because burial in chamber tombs was prohibited, but because there were social groups that did not identify with the normative funeral traditions, and wanted to bury their dead differently.

In addition to the samples deriving from the Lower Citadel, three skeletal individuals from different areas of the Lower Town were also sampled. These individuals were found in funerary contexts of the Early Iron Age (Protogeometric or Geometric period) in the Northwestern (TIR008, TIR013) and Western (TIR020) Lower Town [31].

All individuals from Tiryns included in this study were examined palaeopathologically as far as bone preservation permitted (Table S1). This analysis shows that although the Late Bronze Age population from Tiryns generally had a relatively good state of health, upper and lower respiratory tract disorders were frequent and intense. In the course of a palaeopathological examination, the nasal cavity, paranasal sinuses (upper respiratory tract) and internal rib surfaces (lower respiratory tract: lungs) were examined for vestiges of an inflammatory process (e.g., sinusitis, pleurisy). Such pathologies are most probably multi-factorial, and are certainly linked to exposure to indoor smoke to a large part. Vestiges of a partial yet very pronounced chronic inflammatory process in the upper respiratory tract (e.g., sinusitis) are found in eight (Table S1) out of 11 individuals with sufficient preservation, of which five individuals also show lignite inhalation (TIR001, TIR002, TIR005, TIR006, TIR017). In the area of the lower respiratory tract, five out of 12 individuals (with sufficient bone preservation) showed vestiges of scarring on the inner surfaces of the ribs (pleurisy) and three of them in combination with lignite inhalation (TIR001, TIR006, TIR014). The evidence for lung diseases may possibly be explained by air pollution at the workplace, as well as inside the houses. Thus, the development of chronic respiratory diseases is considerably supported or even causally triggered by exposure of the respiratory air to smoke and the combustion particles contained therein.

Concerning the identification of those industries that may have used lignite as a fuel for pyrotechnologies, there is ample evidence for metalworking dating to the Mycenaean Palatial and post-Palatial Periods in the Lower Citadel [32–37]. Pottery production is also confirmed by the presence of a potter's kiln dating to the very beginning of the post-Palatial Period in the Lower Citadel [38–40], another likely potter's kiln in the Middle Citadel of uncertain Mycenaean date [40,41] and a concentration of potter's kilns of the Early Iron Age (Geometric period) in the Northeastern Lower Town [42]. In a late Palatial workshop in the Lower Citadel, head-shaped composite vessels combining faience, gold embellishments and stone inlays were manufactured [30,33,34,43]. It is unknown where these faience vessels may have been fired. Also attested at Tiryns is the processing of glass [36,44].

2.2 Chania, Greece (sample names "XAN")

The Bronze Age harbour center of ancient Kydonia and its cemeteries are situated under the present-day historic center and suburbs of Chania [45,46]. Ancient Kydonia was involved in long-distance maritime trade exporting oil and other commodities. During LM III, it was a vibrant center of commerce with extensive contacts in the Mediterranean including Sardinia, Italy, Cyprus, Syria, Canaan and Egypt. In LM III, Kydonia became a Mycenaean stronghold, with indications of a palace [47], hints of a military capability able to defend the interests of the administration [48], and local Linear A script was replaced by Linear B. Therefore, Kydonia's population needs to be understood as a multi-lingual and multi-ethnic population of varying origins.

During the Final Palatial period of Kydonia, a necropolis was created over an area of at least 1 sq. km to the south-east of the coastal settlement, beginning at about 50 meters from it. Around 200 tombs have been excavated so far, either isolated burials or arranged in groups. Cut into the marly limestone bedrock, they belong mainly to three discrete architectural types, unknown in Crete before this time, and which replaced the multi-chambered Neopalatial tombs: chamber tombs of Mycenaean type, pit-caves and shaft graves. This fact, combined with the start of a new cemetery and the adoption of new burial practices and customs, implies remarkable social change.

Odos Palama Plot

Seventeen tombs were excavated in 1987 in a building plot at 4 Palama Street under the direction of Maria Andreadaki-Vlazaki and Elpida Hadjidaki. The site is 800m away from the excavated part of the Bronze Age settlement of Kydonia. The tombs are part of a large Late Minoan cemetery extending up to 1500m southeast of the settlement, situated under the suburbs of the modern town of Chania [48].

Tombs of three different types (Fig. S8) had been cut into kouskouros, a soft limestone rock that lay below two to three meters of sand [49]. Besides four chamber tombs (5, 10, 16, 17) that are typical in LM III Crete, there was a simple pit grave (tomb 7), and a 'cavity' (tomb 6), while between these graves and in their vicinity, eleven pit-caves were documented (tombs 1-4, 8,9 and 11-15). The 'caves' were dug into the sides of a deep shaft sunk into the rock. This rare tomb form was previously known only at the LM II–LM III cemetery of Zapher Papoura near Knossos. In 2004, another 32 tombs of this pit-cave type were discovered at 73-77 Igoumenou Gavril Street, all of them richly endowed, furnished with military equipment and dated to LM II–LM III [48]. Similar tombs of Sub-Mycenaean date were also found on Rhodes [50]. Beyond the Aegean, the closest parallels for this tomb type seem to be cave tombs in Cyprus and the Levant.

The Palama Street tombs were used during the LM IIIA2 and IIIB1 [49] and none had been robbed or reused in any way. Grave gifts were few and unexceptional compared to the earlier "warrior tombs" [48]. With the exception of one imported Mycenaean piriform jar, all other 14 pottery vessels were locally made. A circular stone construction may have served some practical or ritual purpose during funeral ceremonies. Connections between this structure and tombs 8 and 15 were demonstrated by joins between shards of coarse and decorated pottery found in all three contexts.

The burial corpus and the social landscape

The 17 tombs yielded a total of 29 inhumation burials, 16 adults (7 males, 9 females) and 13 children, of which one died at perinatal age and seven were under 5 years old. The other five children were found in pit-cave 11 and were between 6 to 11 years of age. All of them appeared to have died in a short space of time, perhaps victims of an epidemic, accident or natural disaster. Two had *cribra orbitalis* caused by iron deficiency anaemia, or perhaps by a heavy parasitic infection.

The mean age at death of males was 34.1 years, and of females 25.6 years. The mean stature of males was 164.54 cm, and of females was 148.83 cm. This difference in male/female stature exceeds the normal 8% difference observed in populations worldwide and has implications for the relative social status of men and women, because stature correlates directly with nutritional status and access to food resources. It is also affected by disease, and factors such as population density, hygiene and the availability of medicines. Caries was 10% higher in females, and the incidence of abscesses and the rate of tooth loss was twice as high in females as males.

The samples analysed are from pit caves and chamber tombs. Tombs 1, 3 and 16 could not be more precisely dated than LM IIIA2–LM IIIB1.

Pit caves

Tomb 1 (XAN001.B) housed a male and a female burial and one undatable sherd. The sample is from the female **1A**. The dentition revealed an abrasive diet, caries and abscesses. The left pubic symphysis gave an age score 23-39 years. The vertebral column exhibited slight lipping on L3, L4 and S1, deemed age or work-related changes.

Tomb 3 (XAN004.B) housed the burial of a young adult female and the rib of a child. Still visible epiphyseal fusion lines of the femur heads and the left ilium indicate an age range of 17-23 years. Pronounced wear on the teeth of the left side of the mouth was probably due to the early development of dental caries and the loss of the lower right molars. Nine teeth had been lost *ante mortem* and six had caries lesions. Nearly all the teeth displayed enamel hypoplasia caused by childhood illness or inadequate nutrition. Vertebral disc herniations, T6-9, and degenerative changes, T10, L4 through S1, are linked with physical stress.

Tomb 8 (XAN002) housed an adult and an embryo in the cave and two adults in the pit. Sherds dated the fill to LM IIIB1. The calculus sample comes from the well-preserved young adult female **8C** sealed in the cave. The petite, gracile skeleton implied a sedentary lifestyle. Five teeth had caries, one had been shed, and there were significant calculus accretions on the incisors. Tooth wear was mild. Incomplete development of a third molar root indicated an age between 18-25 years. The left pubic symphysis suggested an age of 19-20 years. The embryo implicates pregnancy complications or obstructed labour as the likely cause of death.

Three un-Aegean silver signet rings in this tomb were the most exotic finds from the excavation. Two were recovered in the fill of the corridor together with the bones of a man and a woman and one was found with **8C**, the pregnant female. Due to their similarity in size and design, the rings probably originated from the same geographical source. The seal devices of the two best preserved rings present a rather crude quadruped and a human figure. A close parallel, a silver signet ring with the figure of the Egyptian god Bes, guardian of pregnant women and children, was found in Ras Ibn Hani, the harbour town of Ugarit [51].

Most significantly, cluster analysis of morphometric cranial measurements on 20 female populations from mainland Greece, Crete, Cyprus and the Levant, had clustered the females from pit-caves **3** and **8C** between Bronze Age Troy and Middle Bronze Age Ugarit [49]. Although at the time of the 1992 publication no close parallel was available for the rings in tomb 8, these can now be connected with Ugarit.

Chamber tombs

Tomb 10 (XAN003.B), housed a young adult female **10A** and two small children, found with beads, a pendant and a crab's claw. The woman's cranium was stippled with coarse porotic lesions, the aetiology of which could be thalassaemia, a quantitative disorder of the haemoglobin, or some other form of anaemia, such as anaemia associated with chronic infections. Her dental health was poor; 70% of her teeth had caries. Enamel hypoplasia showed she was beleaguered by ill-health in childhood. Fusion of the basilar suture, between 17 and 23 years, was complete. Negligible tooth wear was in the 17-25 year age range. Pronounced development of the right humerus' deltoid muscle contrasted noticeably with the left arm.

Tomb 16 (XAN008) housed two male burials, perhaps secondary burials since the bones were gathered in heaps below the crania with no associated finds. A robust adult male, **16-1,** provided the calculus sample. Calculus on the majority of the teeth was prominent on the buccal surfaces of the lower incisors. The pubic symphysis gave an age estimate of 23-39 years. Synostosis of the cranial sutures suggested an age of 30 to 40 years or more. Pathological changes included degenerative changes to the lower thoracic and lumbar vertebrae (rarely seen today in patients under 60) and plantar fasciitis affecting both heels.

Kouklakis Plot:

In 2004, in the Kouklakis plot at the Mazali site, 60 tombs were excavated by Maria Andreadaki-Vlazaki with the help of Eftyhia Protopapadaki. Among them, quite a few "warrior graves" have been identified. The excavation as a whole is still unpublished and aDNA analysis is ongoing.

Sample XAN 011 derives from the Pit Cave no19 (pit dimensions 2.70 x 1.20 x 2.68 and cave dimensions 2.14 x 1.20 x 2.50 m). Constructed in LM IIIA1, it contained the burial of a warrior, judging by the bronze weapons accompanying him: a sword, a spearhead, a dagger and three small three-handled clay jars.

Based on the study of poorly preserved postcranial remains, this individual appearedto be a young man between 23 to 27 years old. Stature was estimated from a right humerus: 166.53 cm and left femur: 167.15 cm.

Malefakis Plot:

In 2008, two LM III chamber tombs, 1 and 3, and the dromos of a third, tomb 2, were excavated in the Malefakis plot at the junction of Ioannou Sfakianakis and Platonos streets by Efthymia Kataki under the direction of Maria Andreadaki-Vlazaki [52].

Sample XAN 012 derives from Chamber Tomb 3, skeleton 3 within the chamber. Tomb 3 is a subterranean, rock-cut chamber tomb (2.80 x 2.50 x 1.70 m) with a 10 m long dromos. A spherical stirrup jar and fragments of a skyphos with horizontal handles and fishnet decoration, dated to LM IIIB/C, were found in the dromos in disturbed soil at a depth of -2.26m approximately two meters from the tomb's façade. The upper part of the blocking wall of the

chamber had been removed by looters. Bones from two badly preserved burials dated to LM IIIB period were found in the dromos, probably having been disturbed during the robbery.

Inside the almost rectangular chamber, a small part of the roof on the west side had collapsed, but along the perimeter at least four burials were distinguishable. None was in situ, evidently displaced during the robbery. Some burials such as skeleton 2, found in a niche at the northwest corner of the chamber, were secondary interments. Fragments of vases - the foot of an incense burner, the foot of a kylix and part of a stirrup jar with octopus decoration - date to LMIIIB period. A single almond-shaped seal stone with double-sided engraving, portraying a lion and a bird, and two steatite lenticular beads, escaped pillage. In addition to the niche, there were two shallow pits carved in the southeast part of the chamber. The material is being prepared for a detailed publication.

The preliminary study of the Malefaki plot burials is ongoing, and complicated by significant fragmentation and comingling. Burial 3 consists of the comingled remains of two or three individuals. The loose tooth which provided the calculus sample is a lower 1st molar. Identified from photographs, it is presumed to be a left (?) specimen. It cannot be sexed, but wear on the occlusal surface suggests an age range of 17-25 years.

2.3 Alalakh, Turkey (sample names "ALA")

Tell Atchana, Alalakh, is located in the modern state of Hatay, Turkey and was inhabited in the Middle and Late Bronze Age, ca. 2200/2000–1300 BC, with a more ephemeral reoccupation in the Iron Age, ca. 1190–750 BC [53–55]. The Royal Precinct, with palaces, temples, houses as well as administrative and defensive structures, was initially excavated in the 1930s and 1940s by Sir Leonard Woolley [56]. Current excavations under the direction of K. Aslıhan Yener began in 2003. In addition to continuing both the vertical and horizontal exposure of the Royal Precinct (Area 1), the new excavations have uncovered workshops, fortifications, domestic contexts, and two cemetery areas in Areas 2-4. Additional graves have also been found throughout the city in courtyards, abandoned buildings, and under intact floors. In total, 342 graves have been documented, the vast majority of which are simple pit graves with individuals deposited either on their side in the foetal position or on their backs with their legs flexed to one side.

Area 3 revealed an extramural cemetery adjacent to the city's fortification wall, where 134 burials have been excavated to date [57–59]and 33 have been sampled for aDNA. The cemetery was in use from at least the late Middle Bronze Age to the end of the Late Bronze I (ca. 1400 BC), when this area was abandoned. Most of the graves here are single, primary pit graves, although there are a handful of secondary (including perhaps ALA028 and ALA061) and/or multiple burials, as well as three badly preserved infant pot burials. Grave goods are rare in this cemetery, with over half the burials containing no grave goods. When present, they typically consist of one or two vessels (usually a shoulder goblet, a piriform juglet, or a short-neck jar) and perhaps an article of jewelry, most often a metal pin or a beaded bracelet/necklace [58].

The most outstanding feature of this cemetery is the Plastered Tomb, a tomb built of several layers of plaster encasing four individuals that dates to the Late Bronze I [60–62]. ALA001 is the adult male buried in the lowest layer of the tomb, and with him an adult female. Above these individuals, in the upper layer of the tomb, were two additional adults. This is the richest burial found at the site, with 13 vessels and numerous items of adornment, including beads made of

gold, carnelian, and vitreous materials, pins of bronze and silver, and pieces of foil and stamped appliqués made of gold.

The second cemetery is in Area 4, consisting of densely concentrated burials dug into the courtyard and the abandoned remains of several buildings dating to Late Bronze I (ca. 1650/1600–1400 BC). Twenty-six graves have been found here (including ALA125, ALA126, ALA128, and ALA139), and 19 of them have been sampled for aDNA. While most of these are simple pit graves, two cist graves (including ALA126) have been discovered, along with another potentially damaged cist grave (ALA125). These burials are richer in grave goods than those in the extramural cemetery; while the grave goods are of similar types, they appear more frequently, and metal pins are strongly associated with the adult females [63].

The remaining graves were found throughout the city, many in the Royal Precinct, 20 of which have been sampled for aDNA. They date throughout the sequence of occupation, and they are generally the richest in grave goods, comprising a wide variety of imported and local pottery, metal jewelry, and rarer items such as figurines and stone vessels [64].

2.4 Kamid el-Loz, Lebanon (sample names "KEL")

The tell of Kamid el-Loz is located in the Beqa Plain in Lebanon on the central trade axis between the southern Levant and the early urban centers of Syria. It is one of the most important settlement mounds in present-day Lebanon, and has been identified as the city of Kumidi mentioned in Bronze Age textual sources. Archaeological excavations took place under the direction of Rolf Hachmann (University of Saarbrücken) between 1966 and 1981, when the excavations had to be abandoned because of the civil war. Since 1997, excavations have resumed, led by Marlies Heinz (University of Freiburg). The tell was inhabited since the late Neolithic (5th century BC) and flourished during the Middle Bronze Age (2000–1600 BC) and Late Bronze Age (1600–1200 BC). The Late Bronze Age Kamid el-Loz/Kumidi is mentioned several times in Bronze Age texts, e.g. in the so-called 'Amarna Letters' of Pharaoh Amenophis IV/Akhenaten from the 14th century BC, in which Kumidi is mentioned as the seat of the Egyptian governor Puhuru, after Thutmosis III had brought the Beqa Plain under Egyptian rule in the 15th century BC. The most important architectural remains include temples and the palace, all of which were repeatedly destroyed and rebuilt. Outstanding finds were recovered from the so-called treasure house of the Late Bronze Age palace. For our analyses, we selected two individuals from the Middle Bronze Age cemetery which comprised 27 individuals and is situated at the northern slope of the tell [65]. These included:

Grave 99: an adult individual buried together with two ceramic jugs in a rectangular pit.

Grave 100: a well-preserved skeleton together with six ceramic vessels of different shapes in a shallow oval pit with stone lining.

2.5 Megiddo, Israel (sample names "MGD")

The site of Megiddo is located in the Jezreel Valley in Northern Israel. The site has been excavated by four expeditions starting in the early 20th century. The current excavation, led by

Tel Aviv University, is directed by Israel Finkelstein, Mario A.S. Martin and Matthew J. Adams. Megiddo is the type site for the Bronze and Iron Ages in the Levant. It features remains of over 30 settlements which cover the timespan between the Neolithic and the Persian periods (ca. 7th millennium to 4th century BCE). In the Bronze Age, Megiddo was the hub of a city-state which ruled over the western Jezreel Valley. Information on the Late Bronze city-state is provided by the aforementioned Egyptian diplomatic correspondence known as the 'Amarna Letters'. In the Iron Age, Megiddo was an administration centre in the territory of the biblical Northern Kingdom (Israel). It was later the capital of an Assyrian province. The site features an unmatched number of monuments – fortifications, palaces, temples, water systems and other public structures. Samples for this study come from two locations at the site, from layers which date to the late Middle Bronze Age and early Late Bronze Age.

The burials from Megiddo selected for this study were found in Areas H and K. Area H is part of an elite quarter at the north-western edge of the mound. Area K is a more modest residential quarter at the south-eastern edge of the mound. All tombs are intramural burials.

Area H

Tomb 50 is a masonry-constructed chamber tomb in Area H (Level H-16), accessed by a long dromos. It is an elite, perhaps royal tomb, rich in finds.

Tomb 16/H/45 is a double pit burial in Area H (Level H-15), containing two adult males (brothers).

Area K

All burials from Area K belong to Level K-10, and are dated to the MB III-LB I. They were dug in and around a Levantine courtyard house. They include unlined or lined pit burials, a pithos burial and a masonry-constructed chamber tomb for multiple interments.

Tomb 12/K/89 is a pit burial of an adult male.

Tomb 12/K/96 is a double pit burial of two adults (male and female); the deceased were interred next to the foundations of the exterior wall of the courtyard house.

Tomb 14/K/49 is a brick-lined pit burial, sheltering one articulated adult male and a disarticulated adult female.

Tomb 10/K/118 is a pithos burial, containing (mostly partial) skeletal remains of three individuals.

Tomb 14/K/119 Lower is the interment of a juvenile in a stone-lined cist. The deceased was equipped with several vessels, two electrum ear pendants, a necklace of crystal beads, bone inlays of a wooden box and a sheep/goat offering.

Tomb 100 is a masonry-constructed chamber tomb with a corbelled roof in Area K (Level K-10). It contained the disarticulated skeletal remains of a minimum of 23 individuals. Burial offerings contained ceramic vessels (n=61), bronze toggle pins and weapons, scarabs, bone inlays, stone vessels and many other finds.

2.6 Tel Erani, Israel (sample names "ERA")

The site of Tel Erani is located in the Mediterranean coastal plain, ca. 60 km south of modern Tel Aviv-Jaffa. It was excavated as a salvage project of the Israel Antiquities Authority (IAA) [66,67]. Since the 1960s, the site has been excavated to expose important remains mainly of the Early Bronze and Iron Ages [68,69]. Topographically it is divided into an Acropolis and an upper and lower terrace. During the IAA salvage project, an Iron Age Ib-IIa (12–11th century BC) cemetery was excavated on the southern fringe of the lower terrace in two contiguous areas P and Q, with most of the burials found in the latter. Apart from the cemetery, remains of the Iron Age I and II were found in the Acropolis, the uppermost part of the tell, but not dating exactly to the same phases as the cemetery [68].

The Iron Age cemetery comprised at least 43 graves containing 50 individuals (Fig. S9). Most of the individuals were buried in pits dug into the earlier Early Bronze deposits with four phases of burials identified. The bodies were oriented E-W and lay in a supine position; only a few were found lying on their right side. Several individuals were equipped with grave goods mainly comprising pottery vessels, and a few have iron bracelets and flint tools. In two cases, faunal remains of caprines were found in bowls (Liora Kolska Horwitz, pers. comm. 2018). Pairs of jars, one containing a juglet and the other with a bowl placed upside down on top of the jar, were found near the burials, but they do not appear to belong to a specific individual. Several graves contained adults (some identified as female) with babies lying on them with flasks on their hands. The pottery vessels are being studied by Svetlana Talis, IAA.

Seven of the individuals were analyzed by Michal Feldman for aDNA at the Max Planck Institute for the Science of Human History, Jena. Four gave positive results and an additional sample is currently being processed.

The two samples of calculus considered in this paper originated in Layer 3c (3 is equivalent to Iron Age, 'c' means the second stage of the cemetery from bottom to top). Burial 2160 (ERA017) is a 50–60 year old adult male, with at least one premolar lost before death, and another which was carious. The second sample came from Burial 2091 (ERA005), a 30–50 year old adult of unknown sex. Identification of sex and age was carried out on site by Yossi Nagar (IAA), while more detailed work on the dentition is being undertaken by Patricia Smith of The Hebrew University of Jerusalem.

In both individuals the dental wear is severe, with dentine exposed on the greater part of the occlusal surfaces of all teeth. In general, the teeth from the cemetery appear to be similar in size and form to those of other Iron Age populations from the region, but several show unusual pathologies. They include one young individual with an enormous lesion in a second molar, identified as Pre-eruptive intracoronal Resorption (PEIR) in Burial 2151 (also from Layer 3c) [70].

The only radiocarbon date currently available for the cemetery is from skeletal remains in Burial 2141, also from Layer 3c, which gave a date of 1112—1013 cal BC (1 sigma). This and several other samples that are currently being dated were run at the Weizmann Institute, Rehovot by Elisabetta Boaretto and Jamal Ibrahim.

2.7 Abusir el-Meleq, Egypt (sample names "ABU")

The archaeological site Abusir el-Meleq was inhabited from at least 3250 BCE until ca.700 CE. It was a cult center of the Afterlife deity Osiris, located in the Herakleopolite region near the entrance to the Fayum, and had close ties with the surrounding regions such as Fayum and Memphis since at least the 3rd century [71,72]. The cemetery was used from the Predynastic to the Roman Period and was excavated at the beginning of the 20th century by Otto Rubensohn [73]. Rubensohn also found indications of foreign influence at Abusir el-Meleq, e.g. a Greek grave inscription [74]. However, a general issue with this site is the missing archaeological context of the still preserved individuals due to several factors, including incomplete initial documentation and the later destruction and loss of the excavation diaries during the Second World War [75]. Overall, only macerated skulls and mummified heads were preserved, with no additional information such as names or grave goods [74]. There is no possibility of associating the mummified heads with specific burials or grave goods, many of which are now stored in the Ägyptisches Museum in Berlin. The calculus of the two individuals incorporated into this study was chosen based on the radiocarbon date of the respective individuals which shows that their dating falls within the relevant period for this study. Other mummies have already been studied from an archaeogenetic perspective [75].

2.8 Thebes, Egypt (sample name "Horem")

The sample 'Horem.1-5' came from the mummified remains of the named individual Horemkenesi, buried at Deir el-Bahri on the west bank of the River Nile in Thebes (modern Luxor). The site lies at the base of a bay of limestone cliffs in which the goddess Hathor was believed to receive dead souls, and so became both a place of pilgrimage and a high-status burial ground. As early as c.2050 BC, King Montuhotep II constructed his tomb and funerary temple at Deir el-Bahri. The later ruler Hatshepsut (c.1473–1458 BC) also built here, her funerary temple 'Djeser-djeseru' ('most select of places') constructed at Deir el-Bahri, while her tomb lay directly behind this in the adjacent Valley of the Kings. This same alignment also included the large religious complex of the state god Amun at Karnak, which lay directly opposite Deir el-Bahri on the Nile's east bank, and from c.1000 BC Deir el-Bahri became a high status burial ground for Karnak's clergy. This included tomb DB.320, built for one of these priestly families and also used for the mass reburial of royal mummies brought from their original tombs in the Valley of the Kings. The interment of hundreds more Karnak staff within the area's existing tomb and temple structures is also typical of the way in which Egypt's most sacred sites remained highly desirable burial plots into Roman times. Even with Egypt's acceptance of Christianity during the 4th century AD, the site retained its importance, its Arabic name Deir el-Bahri meaning the 'Northern Monastery' which was built over the ancient temples.

With the site gradually covered by rockfalls from the cliffs above, Montuhotep's temple first came to light in 1859 and was eventually cleared by the Egypt Exploration Fund (later Society) between 1903 and 1907 [76]. This work revealed the king's rock-cut burial chamber was fronted by a terraced temple structure, within which a series of shaft tombs contained the burials of the king's daughter and five of his wives who were also priestesses of Hathor [77]. Although their tombs were plundered in antiquity, the women's remains were found in situ. The shaft tomb subsequently numbered 7 (DBXI.7) was excavated at the end of 1904 into 1905 and was found to contain two individuals - the scattered remains of Montuhotep's royal wife Sadhe and a later intrusive burial which lay intact within a painted wooden coffin stylistically of 21st

dynasty date (c.1069-945 BC), still draped in floral garlands. The coffin inscriptions named the mummified occupant as Horemkenesi, a scribe and priest of Amun also known from ancient graffiti - including his own handwriting - found around Deir el-Bahri and in the Valley of the Kings where he had assisted in the removal of the royal mummies from their tombs prior to their reburial at Deir el-Bahri [78]. These same graffiti naming Horemkenesi, his father Huysheri and their known associates also allows Horemkenesi's career to be dated to the very end of the 20th dynasty and beginning of the 21st dynasty, a time of huge political upheaval when the monarchy lost control of southern Egypt to the Karnak priests. Then at his own death "tentatively placed about 1040–1030 BC" [78], Horemkenesi was mummified and buried in the aforementioned shaft tomb 7 (DBXI.7) at Deir el-Bahri. This was in close proximity to the royal mummies he had helped reinter, and within sight of Karnak Temple, where he had worked as a 'wab' (pure) priest responsible for carrying the cult images of the gods in procession.

In the Egyptian authorities' division of finds from the Deir el-Bahri excavations, Horemkenesi's mummy and coffin were given to the Egypt Exploration Fund, who in turn presented them to one of their long-term sponsors Bristol Museum in 1905, with the warning the mummy was already in 'bad preservation [sic]' [78]. Both were then placed on public display until 1976, by which time the body (accession no. Ha.7386) had deteriorated yet further, leading to the decision to unwrap and dissect it in a study described as "the last such investigation to have been carried out in Britain" [78].

Following X-rays in 1978 which placed the age at death at approximately late 50s to 60 years, Horemkenesi's body was unwrapped in the Department of Anatomy at the University of Bristol over a 2 week period in April 1981 [79]. Although the outer layers of linen wrappings contained no evidence of insects, the innermost layers contained large numbers of carrion beetles (*Dermestes frischii, Dermestes ater*) and their larvae, having hatched from eggs laid on and within the corpse which they had then partly consumed, leaving small 'flight holes' in the remaining soft tissue [80]. This explains its poor preservation, exacerbated by the absence of the conifer resins widely used in embalming to counteract such insect damage but missing in the case of Horemkenesi who was embalmed with little more than plant oils and plant waxes [81].

The remaining soft tissue did however reveal that he had been eviscerated as part of the mummification process, his remaining left ear lobe had been pierced, and his face and head had been shaved as was standard practice for the clergy to ensure ritual purity, as represented on Horemkenesi's coffin portraits [79,80]. Unusually, his mouth had been left wide open, revealing worn-down teeth, two of which exhibited caries plus several abscesses [82,83]. There was also a very small amount of calculus, samples of which were taken by Stephen Buckley and Joann Fletcher in the stores of Bristol Museum on 12.10.18.

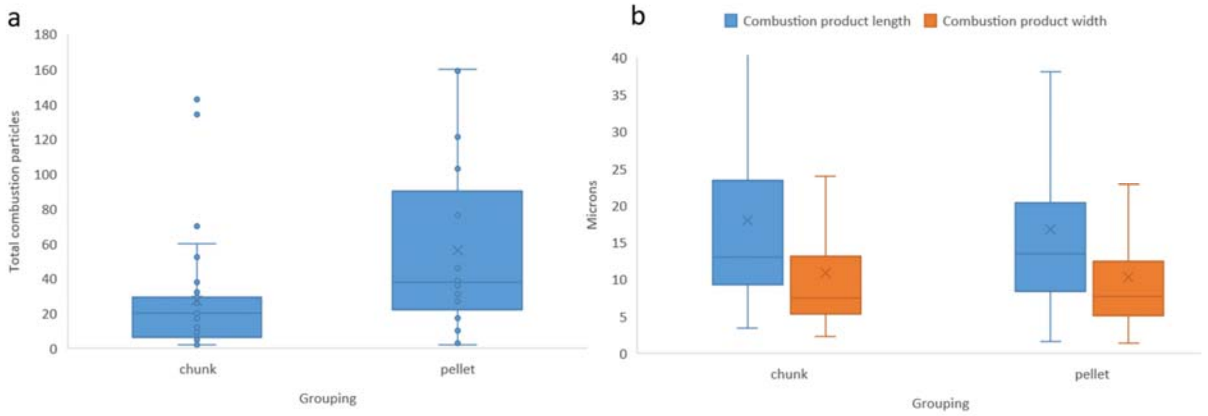

Fig. S1.

Whisker plot showing a) combustion products extracted from the raw dental calculus samples (n=33) and from the residual pellets (n=25) after a prior protein extraction of the dental calculus and b) average length (blue) and width (orange) measurements of the extracted combustion particles from. For some of the samples both residual pellets and chunks were available and both were analyzed. The whisker chart shows an exclusive median. The similarity in combustion particle size demonstrates that there is no major difference when obtained directly from the dental calculus samples compared with the residual pellets after protein extraction, validating their use in this study. Data presented in Table S3.

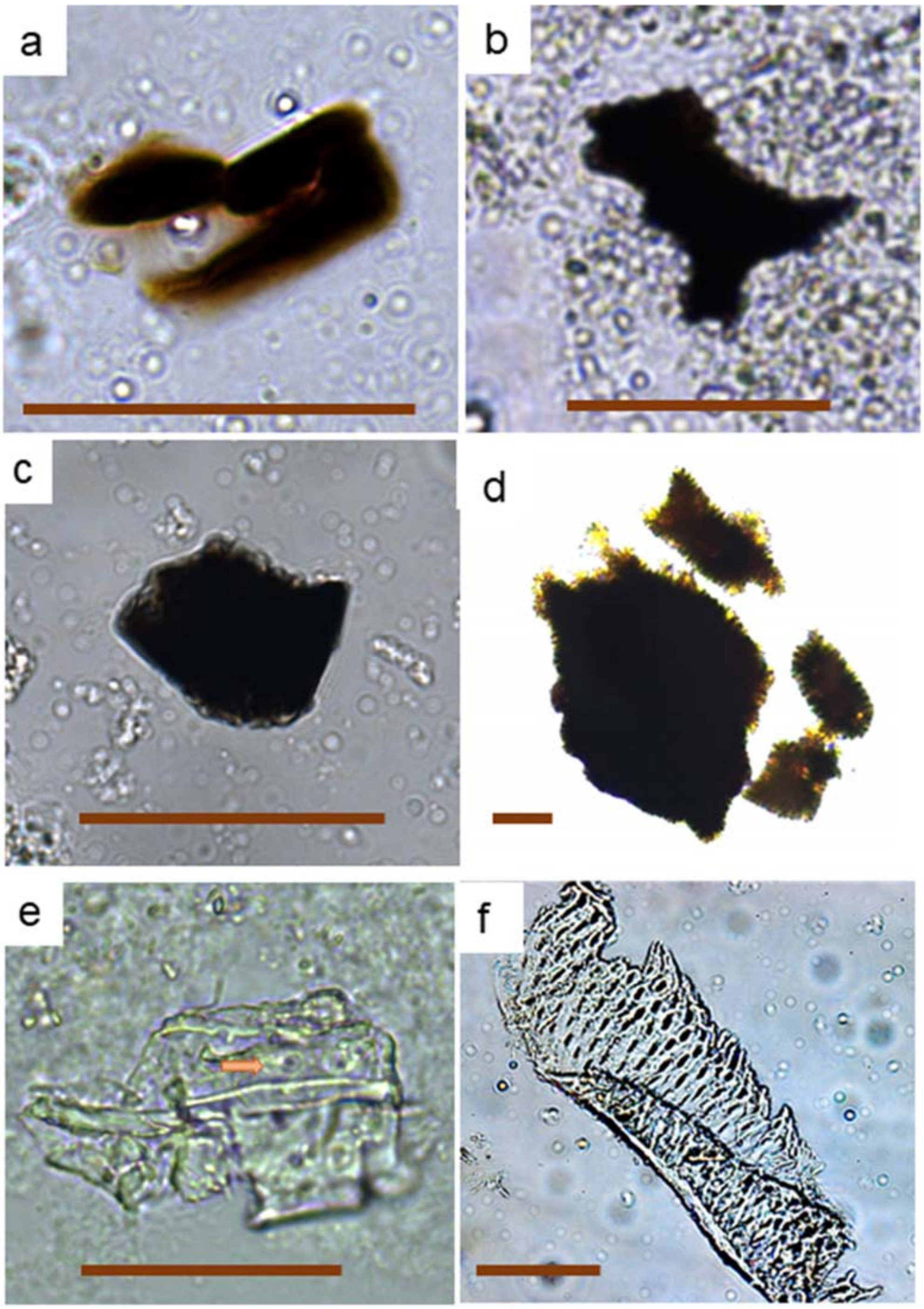

Fig. S2.

A selection of detected microremains. All scale bars represent 50 microns. a-b) combustion particles in TIR003.B, c) smaller indistinct combustion particle in MGD001 from a residual pellet, d) reference lignite particles from Staniantsi (Bulgaria), e) unburnt conifer tissue in TIR001.C, arrow shows bordered pits typical for gymnosperms and f) unburnt wood vessel cf *Alnus* (alder) tissue in ALA004.

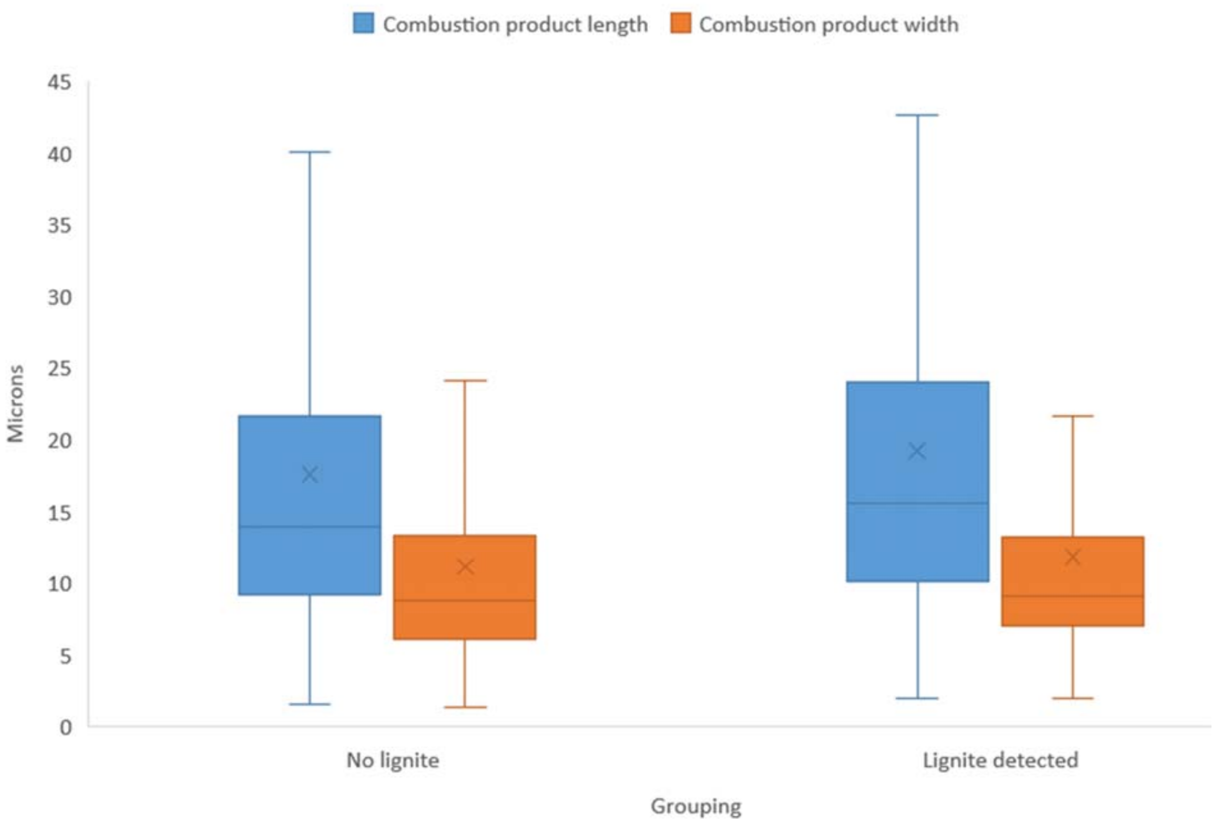

Fig. S3.
Whisker plot showing the mean length (blue) and width (orange) of the combustion particles identified in samples with (n=7) and without chemical evidence (n=45) for the presence of lignite, together with the exclusive median. Due to the large numbers of combustion particles in MGD001, only the first 55 were measured. The whisker plot includes all of the samples tested for microremains (n=54). Not all samples had sufficient material for microremains analysis. Data presented in Table S3.

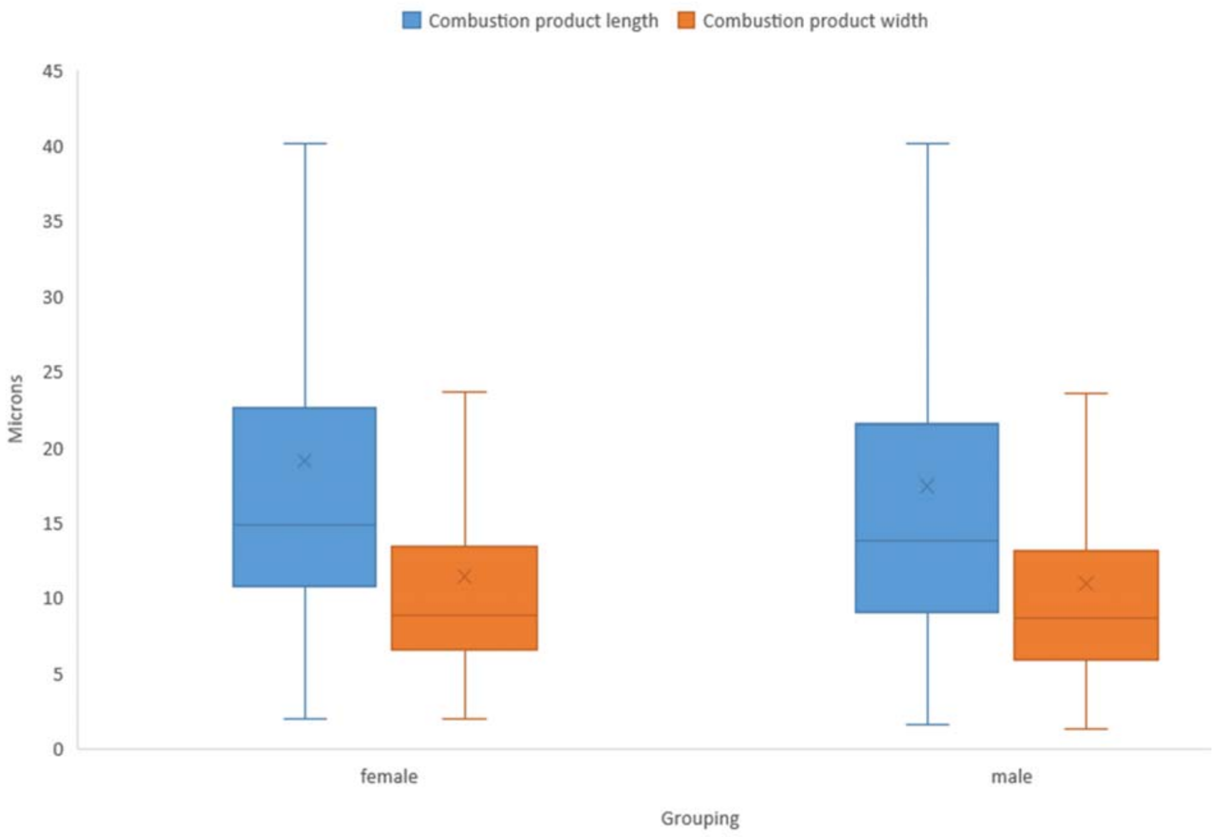

Fig. S4.
Whisker plot comparing the length (blue) and width (orange) of the combustion particles present in male and female individuals included in this study. The exclusive median is marked in the plot. Only individuals for whom biological sex determination was possible were included in this plot (n=40). Data presented in Table S3.

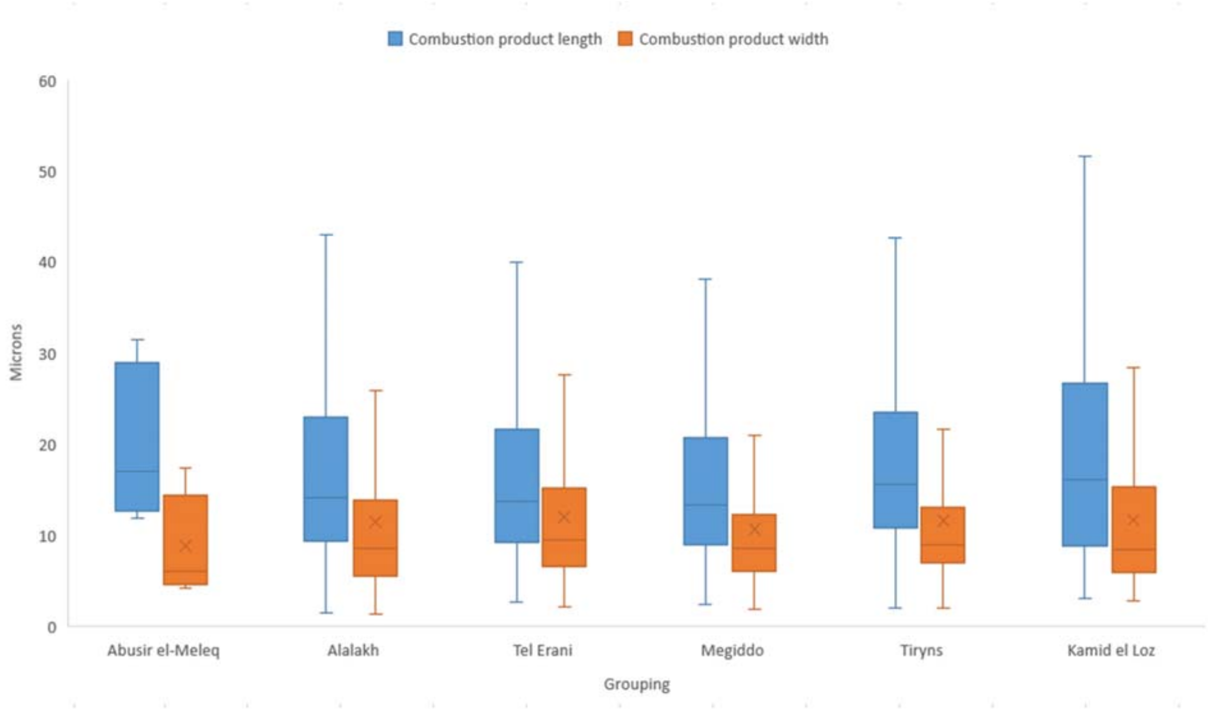

Fig. S5.

Whisker plot comparing the mean length (blue) and width (orange) of the combustion particles present in all of the dental calculus samples studied here from each of the six sites investigated (n=54). The whisker plot includes the exclusive median. Data presented in Table S3.

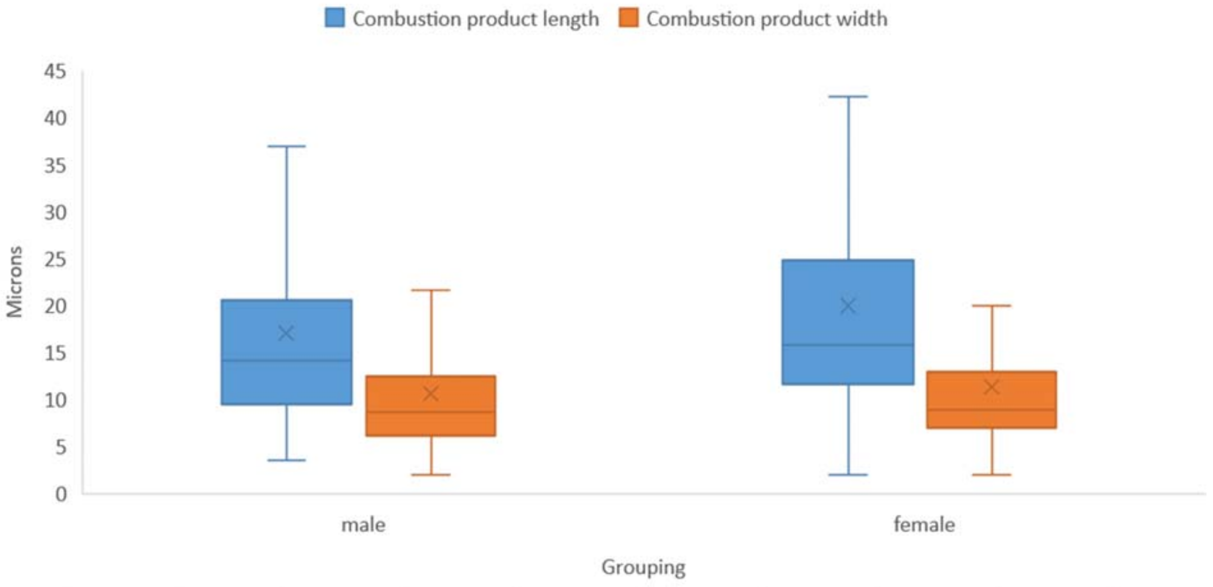

Fig. S6.
Whisker plot comparing the mean length (blue) and width (orange) of the combustion particles identified in the dental calculus of male and female individuals at Tiryns. The whisker plot includes the exclusive median. Biological sex determination was possible for a total of 10 Tiryns individuals. Data presented in Table S3.

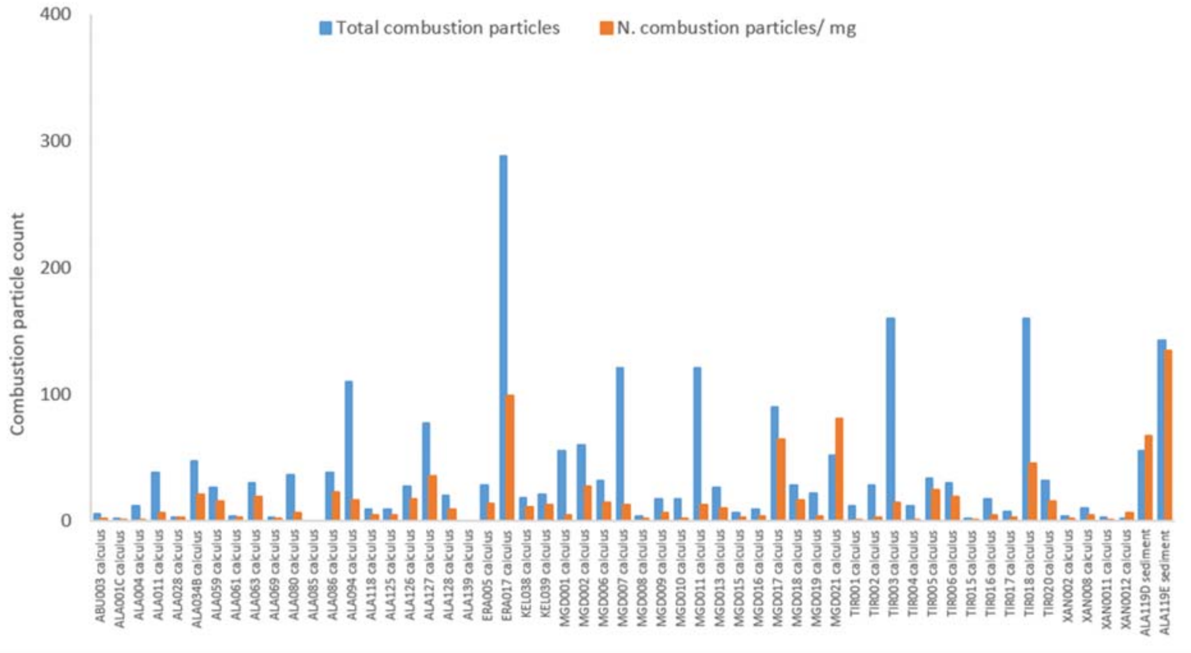

Fig. S7.

Bar chart showing the total number of combustion particles (blue) and the total number of combustion particles per mg of dental calculus (orange) present in all of the dental calculus samples tested in this study (n=54) and in the two sediment control samples (n=2). Data presented in Table S3.

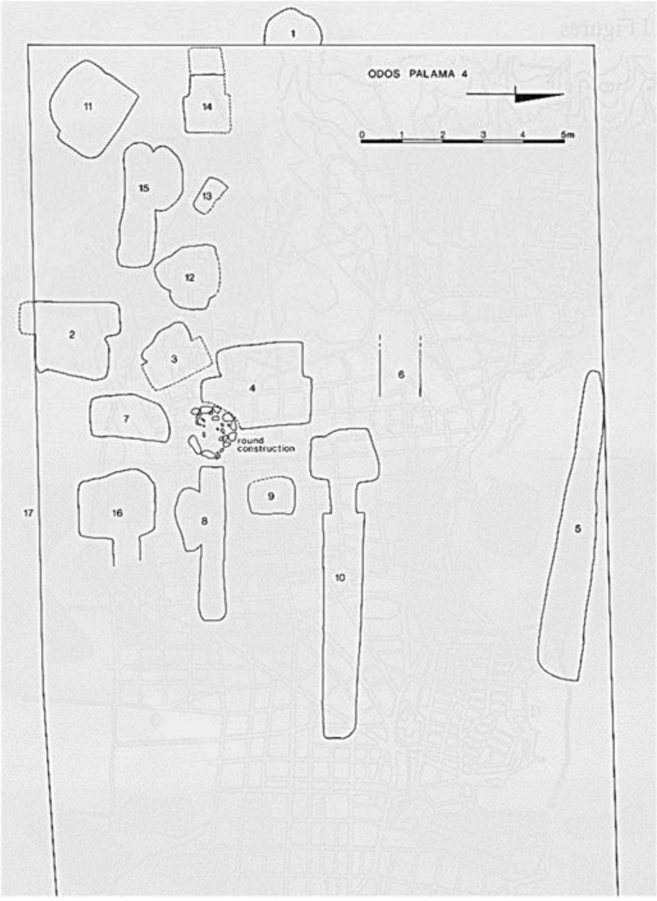

Fig. S8.
Plan of the tombs at 4, Palama Street, Chania.

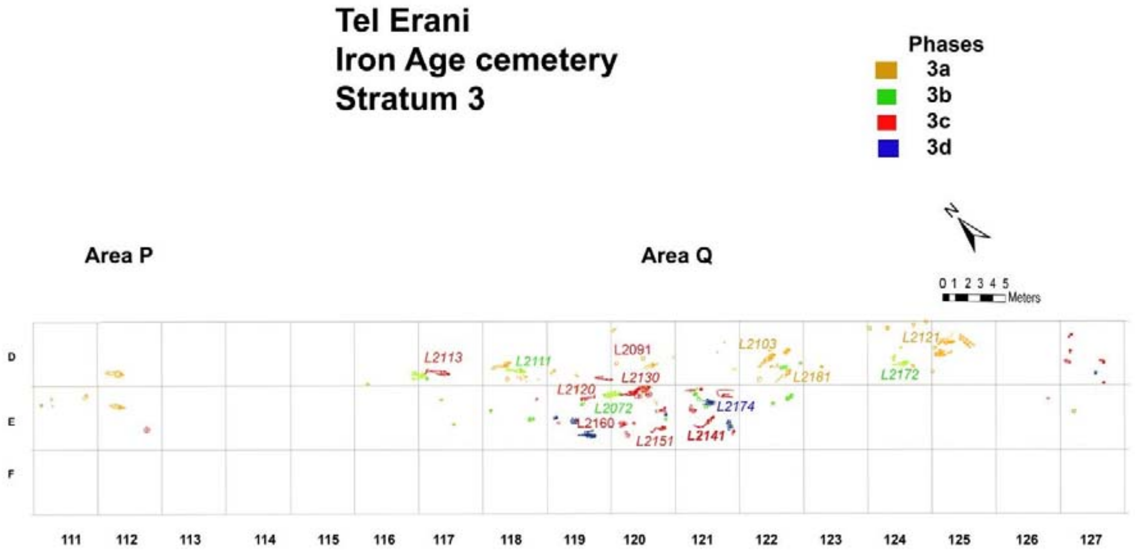

Fig. S9.

Schematic view of the cemetery in Areas P and Q. Numbers in italics are burials from which aDNA samples were taken. The numbers in bold italics indicate the individual that was C14 dated. Regular numbers refer to the burials sampled for dental calculus.

References

1. Scott, A. *et al.* Exotic foods reveal contact between South Asia and the Near East during the second millennium BCE. *PNAS* **118,** e2014956117 (2021).
2. Buckley, S. A., Scott, A. W. & Evershed, R. P. Studies of Organic Residues from Ancient Egyptian Mummies Using High Temperature-Gas Chromatography-Mass Spectronomy and Sequential Thermal Desorption-Gas Chromatography-Mass Spectronomy and Pyrolysis-Gas Chromatography-Mass Spectronomy. *Analyst* **124,** 443–452 (1999).
3. González-Pérez, J. A., Almendros, G., La Rosa, J. M. de & González-Vila, F. J. Appraisal of polycyclic aromatic hydrocarbons (PAHs) in environmental matrices by analytical pyrolysis (Py–GC/MS). *Journal of Analytical and Applied Pyrolysis* **109,** 1–8 (2014).
4. Hardy, K. *et al.* Dental Calculus Reveals Potential Respiratory Irritants and Ingestion of Essential Plant-based Nutrients at Lower Palaeolithic Qesem Cave Israel. *Quaternary International* **398,** 129–135 (2016).
5. Power, R. C., Salazar-García, D. C., Wittig, R. M., Freiberg, M. & Henry, A. G. Dental Calculus Evidence of Taï Forest Chimpanzee Plant Consumption and Life History Transitions. *Scientific Reports* **5,** 1–13 (2015).
6. Power, R. C., Salazar-García, D. C. & Henry, A. G. in *Dolní Věstonice II,* edited by J. A. Svoboda (Academy of Sciences of the Czech Republic; Institute of Archaeology at Brno, 2016), pp. 345–352.
7. Radini, A., Nikita, E., Buckley, S. A., Copeland, L. & Hardy, K. Beyond Food. The Multiple Pathways for Inclusion of Materials Into Ancient Dental Calculus. *American Journal of Physical Anthropology* **162,** 71–83 (2017).
8. Goude, G. *et al.* A Multidisciplinary Approach to Neolithic Life Reconstruction. *Journal of Archaeological Method and Theory* **26,** 537–560 (2019).
9. Power, R. C., Salazar-García, D. C., Straus, L. G., González Morales, M. R. & Henry, A. G. Microremains from El Mirón Cave Human Dental Calculus Suggest a Mixed Plant-Animal Subsistence Economy during the Magdalenian in Northern Iberia. *Journal of Archaeological Science* **60,** 39–46 (2015).
10. Dobney, K. & Brothwell, D. R. in *Scanning Electron Microscopy in Archaeology,* edited by S. L. Olsen (1988), pp. 372–385.
11. Hardy, K. *et al.* Neanderthal Medics? Evidence for Food, Cooking, and Medicinal Plants Entrapped in Dental Calculus. *Naturwissenschaften* **99,** 617–626 (2012).
12. Piperno, D. R. Pytholith and Charcoal Records from Deep Lake Cores in the American Tropics. *MASCA Research Papers in Science and Archaeology* **10,** 59–71 (1993).
13. Juhola, T., Henry, A. G., Kirkinen, T., Laakkonen, J. & Väliranta, M. Phytoliths, Parasites, Fibers, and Feathers from Dental Calculus and Sediment from Iron Age Luistari Cemetery, Finnland. *Quaternary Science Reviews* **222,** 1–8 (2019).
14. Tromp, M., Buckley, H., Geber, J. & Matisoo-Smith, E. EDTA decalcification of dental calculus as an alternate means of microparticle extraction from archaeological samples. *Journal of Archaeological Science: Reports* **14,** 461–466 (2017).
15. Geber, J. *et al.* Relief food subsistence revealed by microparticle and proteomic analyses of dental calculus from victims of the Great Irish Famine. *PNAS* **116,** 19380–19385 (2019).
16. Piperno, D. R. *Phytoliths. A Comprehensive Guide for Archaeologists and Paleoecologists* (Altamira, 2006).

17. Clark, J. S. Particle Motion and the Theory of Charcoal Analysis. Source Area, Transport, Deposition, and Sampling. *Quaternary Research* **30,** 67–80 (1988).
18. Petraco, N. & Kubicki, T. *Color atlas and manual of microscopy for criminalists, chemists, and conservators* (CRC Press, 2004).
19. Rhodes, A. N. A method for the preparation and quantification of microscopic charcoal from terrestrial and lacustrine sediment cores. *The Holocene* **8,** 113–117 (1998).
20. Sander, M. P. & Gee, C. T. Fossil Charcoal. Techniques and Applications. *Review of Palaeobotany and Palynology* **63,** 269–279 (1990).
21. Leys, B. A., Commerford, J. L. & McLauchlan, K. K. Reconstructing Grassland Fire History Using Sedimentary Charcoal. Considering Count, Size and Shape. *PLoS ONE* **12,** 1–15 (2017).
22. Arx, G. v., Crivellaro, A., Prendin, A. L., Cufar, K. & Carrer, M. Quantitative Wood Anatomy. Practical Guidelines. *Frontiers in Plant Science* **7** (2016).
23. Maran, J. in *The Oxford Handbook of the Bronze Age Aegean (ca. 3000-1000 BC),* edited by E. H. Cline (Oxford University Press, 2010), pp. 722–734.
24. Maran, J. in *Mycenaeans Up to Date,* edited by A.-L. Schallin & I. Turnabitu (Svenska Institutet i Athen, 2015), pp. 277–293.
25. Maran, J. in *RA-PI-NE-U,* edited by J. Driessen (UCL, Presses Universitaires de Louvain, 2016), pp. 201–220.
26. Maran, J. *et al.* Tiryns, Griechenland. Die Arbeiten der Jahre 2012 bis 2014. *eDAI-F,* 47–55 (2015).
27. Kilian, K. Ausgrabungen in Tiryns 1982/83. Bericht zu den Grabungen. *AA,* 105–151 (1988).
28. Cavanagh, W. G. & Mee, C. *A Private Place. Death in Prehistoric Greece* (Ȧström, 1998).
29. Kilian, K. Zum Ende der mykenischen Epoche in der Argolis. *JbRGZM* **27,** 166–195 (1980).
30. Maran, J. Forschungen in der Unterburg von Tiryns 2000–2003. Mit einem Beitrag von Peter Marzolff. *AA,* 35–111 (2008).
31. Maran, J. & Papadimitriou, A. Gegen den Strom der Geschichte. Die nördliche Unterstadt von Tiryns: Ein gescheitertes Urbanisierungsprojekt der mykenischen Nachpalastzeit. *AA,* 19-118 (2016).
32. Rahmstorf, L. *Kleinfunde aus Tiryns. Terrakotta, Stein, Bein und Glas/Fayence vornehmlich aus der Spätbronzezeit.* Teilw. zugl.: Heidelberg, Univ., Diss., 2001 (Reichert Verlag, 2008).
33. Brysbaert, A. & Vetters, M. Practicing Identity: A Crafty Deal? *Mediterranean Archaeology and Archaeometry* **10,** 25–43 (2010).
34. Brysbaert, A. & Vetters, M. in *The Mediterranean Mirror,* edited by A. Babbi, F. Bubenheimer-Erhart, B. Marín-Aguilera & S. Mühl (Verlag des Römisch-Germanischen Zentralmuseums, 2015), pp. 161–175.
35. Brysbaert, A., Siozos, P., Vetters, M., Philippidis, A. & Anglos, D. Materials Analyses of Pyrotechnical Objects from LBA Tiryns, Greece, by Means of Laser-Induced Breakdown Spectroscopy (LIBS). Results and A Critical Assessment of the Method. *Journal of Archaeological Science* **83,** 49–61 (2017).
36. Rahmstorf, L. in *Mycenaeans Up to Date.* Current Concepts and New Directions, edited by A.-L. Schallin & I. Turnabitu (Svenska Instituten vid Rom och Athen, 2015). Current Concepts and New Directions, pp. 143–149.

37. Vetters, M., Brysbaert, A., Ntinou, M., Tsartsidou, G. & Margaritis, E. People and Plants. Piecing Together Archaeological and Archaeobotanical Data to Reconstruct Plant Use and Craft Activities in Mycenaean Tiryns. *Opuscula* **9,** 93–132 (2016).
38. Kilian, K. Ausgrabungen in Tiryns 1978, 1979. Bericht zu den Grabungen. *AA,* 149–194 (1981).
39. Prillwitz, S. & Hein, A. in *The Transmission of Technical Knowledge in the Production of Ancient Mediterranean Pottery,* edited by W. Gauß, G. Klebinder-Gauss & C. von Rüden (Österreichisches Archäologisches Institut, 2015), pp. 351–365.
40. Prillwitz, S. unpubl. Dissertation. Universität Heidelberg, 2019.
41. Dragendorff, H. Tiryns. Vorbericht über die Grabungen 1913. *AM* **38,** 329–354 (1913).
42. Maran, J. & Papadimitriou, A. Bericht zu den Ausgrabungen in Stadt-Nordost. Forschungen im Stadtgebiet von Tiryns 1999–2002. *AA,* 99–133 (2006).
43. Kostoula, M. & Maran, J. in *All the Wisdom of the East,* edited by M. Gruber, S. Aḥituv, G. Lehmann & Z. Talshir (Academic Press; Vandenhoeck & Ruprecht, 2012), pp. 193–234.
44. Panagiotaki, M. *et al. in Annales du 16e Congrès de l'Association Internationale pour l'Histoire du Verre, London, 7 - 13 September, 2003.,* edited by M.-D. Nenna (2005), pp. 14–18.
45. Andreadaki-Vlazaki, M. in *The Oxford Handbook of the Bronze Age Aegean (ca. 3000-1000 BC),* edited by E. H. Cline (Oxford University Press, 2010), pp. 518–528.
46. Andreadaki-Vlazaki, M. in *Πεπραγμένα του 10ου Διεθνούς Κρητολογικού Συνεδρίου* (2011), pp. 112–121.
47. Andreadaki-Vlazaki, M. Sacrifices in LM IIIB. Early Kydonia Palatial Centre. *Pasiphae. Rivista di Filologia e Antichità egee* **9,** 27–42 (2015).
48. Andreadaki-Vlazaki, M. (ed.). *Khania (Kydonia). A Tour to Sites of Ancient Memory* (2009).
49. Hallager, B. P. & McGeorge, P. J. P. *Late Minoan III Burials at Khania. The Tombs, Finds and Deceased in Odos Palama* (Åström, 1992).
50. Zervaki, F. in *The "Dark Ages" Revisited,* edited by A. Mazarakis Ainian (Univ. of Thessaly Press, 2011), pp. 769–784.
51. Bounni, A., Lagarce, É. & Lagarce, J. (eds.). *Ras Ibn Hani, I. Le palais nord du bronze récent; fouilles 1979 - 1995, synthèse préliminaire* (Institut Français d'Archéologie du Proche-Orient, 1998).
52. Kataki, E. Οδός Ι. Σφακιανάκη και Πλάτωνος (οικόπεδο Μαλεφάκη). *Archaiologikon Deltion,* 1154–1155 (2008).
53. Yener, K. A. (ed.). *The Amuq Valley Regional Projects 1. Surveys in the Plain of Antioch and Orontes Delta, Turkey, 1995 - 2002* (Oriental Inst. of the Univ. of Chicago, 2005).
54. Yener, K. A. & Ingman, T. (eds.). *Alalakh and Its Neighbours. Proceedings of the 15th Anniversary Symposium at the New Hatay Archaeology Museum, 10-12 June 2015* (Peeters, 2019).
55. Yener, K. A. (ed.). *The Amuq Valley Regional Projects. Excavations in the Plain of Antioch.* The 2003-2004 Excavation Seasons (Ege Yayınları; Koc Universitesi, 2010).
56. Woolley, L. *Alalakh. An Account of the Excavations at Tell Atchana in the Hatay, 1937 - 1949* (Oxford Univ. Press, 1955).
57. Akar, M. in *Questions, Approaches, and Dialogues in Eastern Mediterranean Archaeology,* edited by E. Kozal*, et al.* (Ugarit-Verl, 2017), pp. 215–228.
58. Ingman, T. in *Overturning Certainties in Near Eastern Archaeology,* edited by Ç. Maner, M. T. Horowitz & A. S. Gilbert (Brill, 2017), pp. 245–259.

59. Yener, K. A. & Yazıcıoğlu, G. B. in *The Amuq Valley Regional Projects.* The 2003-2004 Excavation Seasons, edited by K. A. Yener (Ege Yayınları; Koc Universitesi, 2010). The 2003-2004 Excavation Seasons, pp. 11–49.
60. Boutin, A. T. Dissertation. University of Pennsylvania, 2008.
61. Boutin, A. T. in *The Amuq Valley Regional Projects.* The 2003-2004 Excavation Seasons, edited by K. A. Yener (Ege Yayınları; Koc Universitesi, 2010). The 2003-2004 Excavation Seasons, pp. 111–121.
62. Yener, K. A. in *Amilla,* edited by R. B. Koehl (INSTAP Academic Press, 2013), pp. 263–279.
63. Shafiq, R. Evidence of a Possible Elite Cemetery at Alalakh / Tell Atchana. *Arkeometri Sonuçları Toplantıları* **33,** 193–209 (2018).
64. Ingman, T. in *Alalakh and Its Neighbours,* edited by K. A. Yener & T. Ingman (Peeters, 2019), pp. 389–406.
65. Miron, R. in *Bericht über die Ergebnisse der Ausgrabungen in Kāmid el-Lōz in den Jahren 1971 bis 1974,* edited by R. Hachmann (Habelt, 1982), pp. 101–121.
66. Milevski, I., Yegorov, D., Aladjem, E. & Pasternak, M. D. in *Tel Erani I,* edited by K. Ciałowicz, Y. Yekutieli & M. Czarnowicz (Wydawnictow Alter, 2016), pp. 45–57.
67. Yegorov, D. & Milevski, I. Tel 'Erani. *Hadashot Arkheologiyot* **129,** 1888-1900 (2017).
68. Yeivin, S. *First Preliminary Report on the Excavations at Tel "Gat". (Tell Sheykh 'Ahmed el-'Areyny).* Seasons 1956 - 1958 (The Gat Expedition, 1961).
69. Ciałowicz, K., Yekutieli, Y. & Czarnowicz, M. (eds.). *Tel Erani I. A Preliminary Report of the 2013 - 2015 Excavations* (Wydawnictow Alter, 2016).
70. Zilberman, U., Milevski, I., Yegorov, D. & Smith, P. A 3000 Year Old Case of An Unusual Dental Lesion. Pre-eruptive Intracoronal Resorption. *Archives of Oral Biology* **97,** 97–101 (2019).
71. Riggs, C. *The Beautiful Burial in Roman Egypt. Art, Identity, and Funerary Religion* (Oxford Univ. Press, 2005).
72. Falivene, M. R. *The Herakleopolite Nome. A Catalogue of the Toponyms with Introduction and Commentary.* Teilw. zugl.: Diss., 1987 (Scholars Press, 1998).
73. Rubensohn, O. & Knatz, F. Bericht über die Ausgrabungen bei Abusir ell Mäläq im Jahre 1903. *ZÄS* **41,** 1–21 (1904).
74. Welte, B. *Zeitzeugen aus dem Wüstensand. Die altägyptischen Mumienschädel aus Abusir el-Meleq.* Dissertation (Marie Leidorf, 2016).
75. Schuenemann, V. J. *et al.* Ancient Egyptian Mummy Genomes Suggest an Increase of Sub-Saharan African Ancestry in Post-Roman Periods. Supplementary Figures. *Nat Commun* **8** (2017).
76. Naville, E., Hall, H. R. & Ayrton, E. R. *The XIth Dynasty Temple of Deir El-Bahari. Part 1.* Twenty-Eighth Memoir of The Egypt Exploration Fund (1907).
77. Fletcher, J. *The Story of Egypt* (Hodder, 2015).
78. Taylor, J. H. *Unwrapping a Mummy. The Life, Death and Embalming of Horemkenesi* (British Museum Press, 1995).
79. Dawson, D., Giles, S. & Ponsford, M. (eds.). *Horemkenesi. May He Live Forever.* The Bristol Mummy Project (Bristol City Council, 2002).
80. Strong, L. in *Horemkenesi.* The Bristol Mummy Project, edited by D. Dawson, S. Giles & M. Ponsford (Bristol City Council, 2002). The Bristol Mummy Project, pp. 175–176.

81. Buckley, S. A. & Evershed, R. P. in *Horemkenesi.* The Bristol Mummy Project, edited by D. Dawson, S. Giles & M. Ponsford (Bristol City Council, 2002). The Bristol Mummy Project, pp. 179–180.
82. Darling, A. in *Horemkenesi.* The Bristol Mummy Project, edited by D. Dawson, S. Giles & M. Ponsford (Bristol City Council, 2002). The Bristol Mummy Project, pp. 154–155.
83. Leek, F. in *Horemkenesi.* The Bristol Mummy Project, edited by D. Dawson, S. Giles & M. Ponsford (Bristol City Council, 2002). The Bristol Mummy Project, pp. 156–158.
